# Validity of the Intake of Sugars, Amino Acids, and Fatty Acids Estimated Using a Self-administered Food Frequency Questionnaire in Middle-aged and Elderly Japanese: The Japan Public Health Center-based Prospective Study for the Next Generation (JPHC-NEXT) Protocol Area

**DOI:** 10.2188/jea.JE20230132

**Published:** 2024-08-05

**Authors:** Utako Murai, Junko Ishihara, Ribeka Takachi, Ayaka Kotemori, Yuri Ishii, Kazutoshi Nakamura, Junta Tanaka, Hiroyasu Iso, Shoichiro Tsugane, Norie Sawada

**Affiliations:** 1 Division of Cohort Research, National Cancer Center Institute for Cancer Control, Tokyo, Japan; 2 Department of Food and Life Science, School of Life and Environmental Science, Azabu University, Kanagawa, Japan; 3 Department of Food Science and Nutrition, Nara Women’s University Graduate School of Humanities and Sciences, Nara, Japan; 4 Department of Community Preventive Medicine, Niigata University Graduate School of Medical and Dental Sciences, Niigata, Japan; 5 Department of Health Promotion Medicine, Niigata University Graduate School of Medical and Dental Sciences, Niigata, Japan; 6 Institute for Global Health Policy Research, Bureau of International Health Cooperation, National Center for Global Health and Medicine, Tokyo, Japan; 7 Graduate School of Public Health, International University of Health and Welfare, Tokyo, Japan

**Keywords:** validation study, sugar, amino acid, fatty acid, Japan

## Abstract

**Background:**

The Japanese database of food composition was revised in 2020, during which both the number of food items and the number of food items measured for sugars, amino acids, and fatty acids were increased. We evaluated the validity of estimated intakes of sugars, amino acids and fatty acids using a long food frequency questionnaire (long-FFQ) among middle-aged and elderly Japanese.

**Methods:**

From 2012 to 2013, 240 men and women aged 40–74 years from five areas in the JPHC-NEXT protocol were asked to respond to the long-FFQ and provide a 12-day weighed food record (WFR) as reference. The long-FFQ, which included 172 food and beverage items and 11 seasonings, was compared with a 3-day WFR, completed during each distinct season, and validity was assessed using Spearman’s correlation coefficients.

**Results:**

Percentage differences based on the long-FFQ with the 12-day WFR in men and women varied from −84.4% to 419.6%, and from −75.8% to 623.1% for sugars, −17.5% to 3.8% and −5.8% to 19.6% for amino acids, and −58.5% to 78.8% and −43.4% to 129.3% for fatty acids, respectively. Median values of correlation coefficients for the long-FFQ in men and women were 0.52 and 0.42 for sugars, 0.38 and 0.37 for amino acids, and 0.42 and 0.42 for fatty acids, respectively.

**Conclusion:**

The long-FFQ provided reasonable validity in estimating the intakes of sugars, amino acids, and fatty acids in middle-aged and elderly Japanese. Although caution is warranted for some nutrients, these results may be used in future epidemiological studies.

## INTRODUCTION

Recently, the association of specific nutrient types, such as sugars, amino acids, and fatty acids, with chronic diseases has been studied using observational studies in Western countries.^1^^–^^6^ To examine these associations in Asian populations, however, it is important to confirm the validity of nutrient intake estimations from food frequency questionnaires (FFQs). FFQs represent one of the most commonly used methods of estimating daily dietary intake and are generally used in epidemiological studies. In addition, fatty acids and sugars are also used to evaluate the dietary quality of foods, such as ultra-processed foods,^7^ and confirming the validity of their intake will also useful in assessing the quality of diet, such as dietary patterns.

In Japan, the Food Composition Table was re-issued in 2020 (FCT 2020), during which the number of food items measured for sugar, amino acid, and fatty acid was increased.^8^ The FCT 2020 also included updated nutrition calculations for the utilization of energy-producing components,^9^ in which energy is calculated using component values and energy conversion factors based on a method recommended by the Food and Agriculture Organization of the United Nations (FAO).^10^^,^^11^

The Japan Public Health Center-based Prospective Study for the Next Generation (JPHC-NEXT) study is an ongoing large-scale follow-up study initiated in Japan in 2011. The study uses a FFQ. The JPHC-NEXT study previously reported that correlation coefficients for energy and major nutrients between the FFQ and weighed food records (WFR) were moderate or high for many nutrients, including protein, fat, carbohydrates, vitamins, and minerals.^12^ Even prior to this, estimated dietary intakes of amino acids and sugar using the FFQ were examined in the former Japan Public Health Center-based Prospective (JPHC) study conducted in 1995,^13^^,^^14^ and validity was found to be good or acceptable. However, these validations of sugar and amino acid intake were conducted using earlier versions of the food composition tables, namely FCT 2015 and FCT 2010, respectively. Moreover, they have not been validated in the JPHC-NEXT study population, which is a different population from the JPHC study. Indeed, the Japanese FCT for substituted fatty acids was developed mainly using the 4th revised edition of the FCT published in 1982 and a FCT of fatty acids published in 1990.^15^ The FCT for fatty acids has been updated several times since then, and estimated fatty acid intake in this study population should accordingly be re-validated using the updated FCT.

The aim of this study was to validate the intakes of sugars, amino acids, and fatty acids between the FFQ and WFR in the JPHC-NEXT Protocol Area using the FCT 2020. In addition, because the calculation of energy intake has been updated and the number of food items measured for several major nutrients increased, we also confirmed energy and major nutrients using the updated food composition tables.

## METHODS

### Study settings and participants

The JPHC-NEXT protocol area included a total of 255 participants from five areas of Japan: Yokote, Saku, Chikusei, Murakami, and Uonuma. Details of the study design have been described elsewhere.^12^ In this study, we included 240 subjects (98 men and 142 women) aged 40–74 years who were eligible for analysis. The study was approved by the Institutional Review Board of the National Cancer Center, Japan and all other collaborating research institutions. All participants provided written informed consent to participate in the JPHC-NEXT protocol area.

### Data collection

Between November 2012 and December 2013, reference intake data were obtained from all participants using the 3-consecutive-day WFRs over four seasons (12-day WFR) at intervals of approximately 3 months. We used information from the long-FFQ in December 2013. In addition, 228 of these subjects (92 men and 136 women) also answered the short-FFQ in February 2014. Information on height, weight, physical activity, smoking, and drinking status was collected using a self-reported questionnaire. We categorized excessive drinkers by ≥280 g ethanol/week for men and ≥140 g ethanol/week for women using definition to promote public health in Japanese policy.^16^

### Weighed food records and food frequency questionnaire

The 12-day WFR and long- and short-FFQs in this study have been described in detail elsewhere.^12^ Intakes of energy and total 153 nutrients, including 31 sugars, 21 amino acids and 47 fatty acids, were calculated using the Standard Tables of Food Composition 2020 in Japan,^8^ as well as purpose-developed food composition tables for isoflavones and lycopene in Japanese foods.^17^^,^^18^ In a previous study,^12^ water-soluble and -insoluble dietary fiber was measured using the Prosky or modified Prosky method (based on the Association of Official Analytical Chemists [AOAC] 985.29 method) using FCT 2010. In FCT 2020, dietary fiber was additionally measured using the AOAC.2011.25 method, which also quantifies low-molecular-weight water-soluble dietary fiber, and this value was included as part of total dietary fiber. In FCT 2020, values for available carbohydrates (monosaccharide equivalents) which were unmeasured in 624 of the 2,478 food items were substituted by values of available carbohydrates (calculated by difference) and multiplied by 4/3.75 to convert to respective monosaccharide equivalents. Similarly, available carbohydrates (mass matter) unmeasured in 637 of 2,478 food items were also substituted by value of available carbohydrates (calculated by difference). These correspondences were developed in consideration of the impact of using the FCT 2020 in Japan.^19^

### Statistical analysis

The mean intake of each nutrient, estimated using the long-FFQ, was compared to intakes estimated using the 12-day WFR in 98 men and 142 women. Percentage differences in mean intake were calculated using the following formula: (long-FFQ mean − WFR mean)/WFR mean × 100. To determine validity, Spearman’s rank correlation coefficients (CCs) between intakes based on the FFQs and 12-day WFR were calculated as energy-adjusted values using the residual model.^20^ The energy adjustment for WFR was conducted after calculating the average for 12 days. We corrected the observed CCs for the attenuating effect of random intra-individual error based on the usual intake of energy and nutrients.^20^ The deattenuated value was corrected using the ratios of the intra- to inter-individual variances based on each crude nutrient intake in 12-day WFR according to the following formula:

deattenuated CCx=energy-adjusted CCx∗SQRT(1+λx/n),

where the observed energy-adjusted CCx is the correlation in energy-adjusted value for nutrient x, λx is the ratio of intra- to inter-individual variance, and n is the number of dietary records (12 days).^20^ Additionally, we evaluated the validity of the short-FFQ in 92 men and 136 women using the same statistical methods. The short-FFQ has fewer questionnaires than the long-FFQ and does not evaluate gluconic acid, chlorogenic acid, or quinic acid. We calculated those items in the long-FFQ included in the FCT 2020. Although we reported the reliability for nutrients in the previous study,^21^ we did not consider intra-class correlation (ICC), in which a higher value indicates lower within-person variation. The ICC statistics were therefore calculated for each nutrient separately to determine the degree of similarity between this long-FFQ and the same long-FFQ conducted a year ago. ICC was analyzed using the SAS MIXED procedure. In accordance with the criteria of Lombard et al,^22^ percent differences ≤10.0 were categorized as good, 11.0–20.0 as acceptable, >20.0 as poor, and CCs between dietary intakes estimated by the FFQ and by 12-day WFR were evaluated with ≥0.50 categorized as good, 0.20–0.49 as acceptable, and <0.20 as poor. All analyses were performed using SAS Version 9.4 (SAS Institute Inc., Cary, NC, USA).

## RESULTS

Table 1 shows the basic characteristics of 240 subjects who completed the long-FFQ, as reproduced from a previous study.^12^ Mean age was 57.4 years in men and 57.0 years in women, and mean body mass index (BMI) was 23.7 (standard deviation [SD], 2.8) in men and 22.8 (SD, 3.1) in women. In most areas, the proportion of women was higher than that of men. Physical activity (metabolic equivalent) was similar between men and women. The proportion of current smokers and excessive drinkers was 26.5% and 39.8% in men and 2.1% and 4.9% in women, respectively.

**Table 1.** Subject characteristics (98 men and 142 women)

	Men	Women
Area		
Yokote in Akita, *n*	28	37
Saku in Nagano, *n*	22	33
Chikusei in Ibaraki, *n*	15	33
Uonuma in Niigata, *n*	16	16
Murakami in Niigata, *n*	17	23
Age,^a,b^ years	57.4 (8.6)	57.0 (8.6)
40–64 years, %	40.8	59.2
65–74 years, %	40.8	59.2
Body height,^a,b^ cm	168.2 (6.8)	156.6 (5.7)^**^
Body weight,^a,b^ kg	67.0 (9.3)	55.9 (8.0)^**^
BMI,^a,b^ kg/m^2^	23.7 (2.8)	22.8 (3.1)^*^
Physical activity,^a,b^ MET-h/day	38.6 (8.6)	38.8 (6.6)
Current smoker,^b^ %	26.5	2.1^**^
Excessive drinker,^b,c^ %	39.8	4.9^**^

BMI, body mass index; MET, metabolic equivalent.

^a^Values are reported as mean (standard deviation).

^b^*P*-values refer to Student’s *t*-test or Chi-square test between sex for each; ^*^P < 0.05, ^**^P < 0.01.

^c^≥280 g ethanol/week in men and ≥140 g ethanol/week in women to prevent lifestyle-related diseases according to Ministry of Health, Labour and Welfare in Japan (https://www.mhlw.go.jp/www1/topics/kenko21_11/b5.html; accessed 26.06.2023).

### Validity of the long-FFQ for mean intakes of sugar, amino acid, and fatty acid

Table 2A, Table 2B, and Table 2C show the percentage differences and their correlations for intakes of sugars, amino acids, and fatty acids, respectively, as assessed by the long-FFQ and 12-day WFR for men and women. Percentage differences based on the long-FFQ with the 12-day WFR in men and women varied from −84.4% (mannitol) to 419.6% (fumaric acid) and −75.8% (mannitol) to 623.1% (fumaric acid) for sugars; −17.5% (hydroxyproline) to 3.8% (proline) and −5.8% (hydroxyproline) to 19.6% (proline) for amino acids; and −58.5% (icosatrienoic acid [n-3]) to 78.8% (heptanoic acid) and −43.4% (icosatrienoic acid [n-3]) to 129.3% (heptanoic acid) for fatty acids, respectively. Percentage differences for each nutrient in the short-FFQ showed similar tendencies to those in the long-FFQ (eTable 2A, eTable 2B, and eTable 2C).

**Table 2A.** Daily intakes of sugars according to the long-FFQ, and percentage differences between intakes by the long-FFQ and 12d-WFR and their correlations in men and women aged 40–74 years

	Men (*n* = 98)					Women (*n* = 142)					
	
	12d-WFR	Long-FFQ	%diff^a^	CC^b^	CC^c,d^	12d-WFR	Long-FFQ	%diff^a^	CC^b^	CC^c,d^	Number of items^e^
	
	Mean (SD)	Mean (SD)				Mean (SD)	Mean (SD)				
Available carbohydrate; monosaccharide equivalents, g	302.9 (62.6)	322.4 (103.3)	6.4^*^	0.69	0.72^**^	247.8 (39.7)	281.5 (85.9)	13.6^**^	0.39	0.41^**^	175
Available carbohydrate; mass matter, g	279.6 (57.7)	297.1 (95.1)	6.3^*^	0.69	0.72^**^	229.6 (37.0)	260.6 (80.2)	13.5^**^	0.39	0.41^**^	175
Available carbohydrate; calculated by difference, g	285.6 (59.2)	307.2 (98.3)	7.6^*^	0.68	0.71^**^	234.6 (37.0)	270.2 (83.0)	15.2^**^	0.38	0.40^**^	177
Starch, g	187.2 (47.2)	206.8 (76.4)	10.5^**^	0.60	0.63^**^	137.2 (27.4)	162.1 (43.6)	18.2^**^	0.42	0.45^**^	57
Glucose, g	11.7 (5.0)	11.0 (7.2)	−5.7	0.60	0.64^**^	10.9 (4.0)	12.4 (8.6)	13.6^*^	0.51	0.55^**^	73
Fructose, g	9.0 (4.5)	11.5 (7.6)	27.8^**^	0.49	0.52^**^	10.2 (4.4)	15.4 (10.9)	51.0^**^	0.52	0.57^**^	67
Galactose, g	0.3 (0.3)	0.5 (1.3)	51.9	0.56	0.58^**^	0.4 (0.3)	0.9 (1.6)	123.4^**^	0.73	0.77^**^	3
Sucrose, g	21.7 (11.7)	15.1 (10.6)	−30.5^**^	0.67	0.71^**^	27.8 (10.0)	22.6 (14.6)	−18.8^**^	0.43	0.43^**^	69
Maltose, g	0.9 (0.5)	0.8 (0.7)	−13.3	0.49	0.61^**^	1.1 (0.5)	0.9 (0.8)	−15.5^*^	0.40	0.65^**^	19
Lactose, g	4.4 (3.6)	7.6 (9.7)	73.3^**^	0.55	0.58^**^	6.2 (4.3)	12.4 (14.0)	100.3^**^	0.48	0.50^**^	12
Trehalose, g	0.2 (0.2)	0.2 (0.2)	−23.1^**^	0.24	0.34^*^	0.2 (0.1)	0.2 (0.2)	17.5^*^	0.26	0.38^**^	6

Total dietary fiber,^f^ g	24.3 (6.9)	17.7 (8.5)	−27.2^**^	0.54	0.57^**^	21.9 (5.8)	20.7 (9.7)	−5.5	0.52	0.54^**^	97
Total dietary fiber (AOAC.2011.25),^g^ g	10.9 (3.4)	5.8 (4.2)	−46.7^**^	0.12	0.14	8.1 (2.2)	5.4 (3.3)	−33.2^**^	−0.03	−0.03	10
Low-molecular-weight water-soluble dietary fiber,^h^ g	4.6 (1.5)	1.7 (1.3)	−63.1^**^	0.04	0.04	3.2 (0.9)	1.4 (0.8)	−56.8^**^	−0.08	−0.09	10
High-molecular-weight water-soluble dietary fiber,^i^ g	1.7 (0.9)	1.7 (1.3)	−0.1	0.36	0.44^**^	1.4 (0.6)	1.6 (0.9)	15.6^*^	0.16	0.23	10
Water-insoluble dietary fiber,^j^ g	4.7 (1.6)	2.5 (1.7)	−47.5^**^	0.12	0.13	3.6 (1.0)	2.5 (1.6)	−31.0^**^	0.03	0.04	10
Resistant starch, g	0.7 (0.2)	0.4 (0.3)	−38.0^**^	0.42	0.47^**^	0.5 (0.2)	0.5 (0.6)	−14.2	0.25	0.30^**^	7
Sorbitol, g	0.3 (0.3)	0.3 (0.3)	−0.2	0.56	0.72^**^	0.4 (0.4)	0.5 (0.4)	14.2	0.51	0.61^**^	5
Mannitol, g	0.17 (0.21)	0.03 (0.03)	−84.4^**^	−0.05	−0.08	0.16 (0.19)	0.04 (0.03)	−75.8^**^	0.15	0.23	4

Acetic acid, g	0.3 (0.2)	0.1 (0.1)	−72.1^**^	0.33	0.36^**^	0.2 (0.1)	0.1 (0.1)	−52.8^**^	0.08	0.10	6
Lactic acid, g	0.5 (0.4)	0.7 (1.3)	45.9	0.51	0.54^**^	0.5 (0.3)	1.1 (1.4)	97.1^**^	0.66	0.70^**^	9
Gluconic acid, g	0.001 (0.003)	0.001 (0.003)	−37.8	0.11	0.13	0.001 (0.003)	0.002 (0.004)	40.9	0.24	0.37^**^	1
Oxalic acid, g	0.10 (0.08)	0.08 (0.09)	−12.8	0.38	0.56^**^	0.10 (0.09)	0.11 (0.14)	9.8	0.31	0.43^**^	4
Succinic acid, g	0.017 (0.065)	0.010 (0.043)	−43.8	0.40	0.44^**^	0.007 (0.020)	0.005 (0.018)	−28.8	0.22	0.32^**^	1
Fumaric acid, g	0.001 (0.002)	0.004 (0.004)	419.6^**^	0.13	0.18	0.001 (0.001)	0.005 (0.005)	623.1^**^	0.15	0.28	1
Malic acid, g	0.5 (0.3)	0.4 (0.3)	−7.8	0.60	0.65^**^	0.5 (0.3)	0.7 (0.4)	26.4^**^	0.52	0.56^**^	29
Tartaric acid, g	0.03 (0.08)	0.05 (0.08)	38.3	0.44	0.51^**^	0.03 (0.04)	0.06 (0.07)	122.2^**^	0.26	0.44^**^	2
Citric acid, g	0.8 (0.4)	0.8 (0.7)	1.7	0.52	0.58^**^	0.9 (0.4)	1.2 (0.9)	33.8^**^	0.38	0.42^**^	29
Ferulic acid, mg	1.4 (1.4)	1.2 (1.2)	−14.6	0.31	0.37^**^	1.5 (1.2)	1.6 (1.7)	5.2	0.34	0.48^**^	3
Chlorogenic acid, mg	0.1 (0.2)	0.1 (0.2)	−1.8	0.18	0.28	0.1 (0.3)	0.1 (0.2)	−11.8	0.21	0.39^*^	1
Quinic acid, g	0.03 (0.03)	0.03 (0.03)	12.7	0.25	0.41^*^	0.05 (0.06)	0.07 (0.08)	39.9^**^	0.39	0.49^**^	3

12d-WFR, 12-day weighed food record; CC, correlation coefficient; FFQ, food frequency questionnaire; SD, standard deviation.

^a^Percentage differences: (FFQ-12d-WFR)/12d-WFR × 100 (%). *P*-values refer to the paired *t*-test between intakes by long-FFQ and those by 12d-WFR for each; ^*^P < 0.05, ^**^P < 0.01.

^b^Spearman’s rank correlation coefficients based on energy-adjusted values.

^c^Spearman’s rank correlation coefficients based on energy-adjusted values and expressed as deattenuated CC. ^*^P < 0.05, ^**^P < 0.01.

^d^Deattenuated CCx = energy-adjusted CCx × SQRT(1 + λx/n), where λx is the ratio of within- to between-individual variance for nutrient x and n is the number of dietary records (12 days).

^e^Number of items in the long-FFQ covered by the Food Composition Table 2020 of Japan.

^f^Total dietary fiber was derived by combination of the AOAC.2011.25 method with either the Prosky or modified Prosky method.

^g^Total dietary fiber (AOAC.2011.25) measured using only the AOAC.2011.25 method.

^h^Low-molecular-weight water-soluble dietary fiber that remains soluble in 78% aqueous ethanol.

^i^High-molecular-weight water-soluble dietary fiber that precipitates from 78% aqueous ethanol.

^j^Water-insoluble dietary fiber measured using only the AOAC.2011.25 method.

**Table 2B.** Daily intakes of amino acids according to the long-FFQ, and percentage differences between intakes by the long-FFQ and 12d-WFR and their correlations in men and women aged 40–74 years

	Men (*n* = 98)					Women (*n* = 142)					
	
	12d-WFR	Long-FFQ	%diff^a^	CC^b^	CC^c,d^	12d-WFR	Long-FFQ	%diff^a^	CC^b^	CC^c,d^	Number of items^e^
	
	Mean (SD)	Mean (SD)				Mean (SD)	Mean (SD)				
Isoleucine, mg	3,472 (814)	3,412 (1,479)	−1.7	0.36	0.38^**^	2,932 (626)	3,360 (1,372)	14.6^**^	0.34	0.36^**^	153
Leucine, mg	6,128 (1,403)	6,100 (2,567)	−0.5	0.34	0.36^**^	5,172 (1,077)	5,975 (2,406)	15.5^**^	0.33	0.35^**^	153
Lysine, mg	5,253 (1,350)	4,899 (2,306)	−6.7	0.36	0.39^**^	4,384 (1,056)	4,920 (2,203)	12.2^**^	0.33	0.36^**^	153
Methionine, mg	1,841 (435)	1,774 (761)	−3.6	0.32	0.34^**^	1,508 (333)	1,714 (708)	13.7^**^	0.32	0.35^**^	152
Cystine, mg	1,193 (250)	1,234 (485)	3.4	0.40	0.42^**^	989 (182)	1,141 (404)	15.3^**^	0.37	0.40^**^	152
Sulfur-containing amino acids, mg	3,049 (682)	3,007 (1,233)	−1.4	0.34	0.36^**^	2,506 (512)	2,859 (1,103)	14.1^**^	0.34	0.36^**^	153
Phenylalanine, mg	3,617 (802)	3,626 (1,494)	0.3	0.37	0.39^**^	3,061 (610)	3,507 (1,354)	14.5^**^	0.37	0.40^**^	153
Tyrosine, mg	2,902 (669)	2,925 (1,253)	0.8	0.38	0.40^**^	2,447 (515)	2,866 (1,162)	17.1^**^	0.35	0.37^**^	153
Aromatic amino acids, mg	6,535 (1,473)	6,573 (2,765)	0.6	0.37	0.39^**^	5,522 (1,130)	6,394 (2,525)	15.8^**^	0.36	0.38^**^	153
Threonine, mg	3,422 (816)	3,275 (1,424)	−4.3	0.35	0.37^**^	2,856 (629)	3,203 (1,314)	12.1^**^	0.36	0.38^**^	153
Tryptophan, mg	976 (224)	971 (409)	−0.6	0.39	0.41^**^	822 (171)	947 (375)	15.2^**^	0.39	0.41^**^	152
Valine, mg	4,169 (952)	4,139 (1,729)	−0.7	0.36	0.38^**^	3,509 (732)	4,056 (1,620)	15.6^**^	0.34	0.37^**^	153
Histidine, mg	2,605 (651)	2,539 (1,077)	−2.5	0.34	0.37^**^	2,105 (490)	2,439 (1,037)	15.8^**^	0.34	0.37^**^	153
Arginine, mg	5,058 (1,176)	4,870 (1,971)	−3.7	0.42	0.44^**^	4,151 (922)	4,564 (1,751)	10.0^**^	0.46	0.49^**^	153
Alanine, mg	4,160 (988)	3,945 (1,605)	−5.2	0.34	0.36^**^	3,387 (759)	3,730 (1,462)	10.1^**^	0.40	0.43^**^	153
Aspartic acid, mg	7,548 (1,837)	7,270 (3,108)	−3.7	0.44	0.46^**^	6,366 (1,455)	7,110 (2,855)	11.7^**^	0.46	0.49^**^	153
Glutamic acid, mg	15,033 (3,185)	14,730 (6,059)	−2.0	0.33	0.35^**^	12,847 (2,379)	14,377 (5,592)	11.9^**^	0.28	0.30^**^	153
Glycine, mg	3,823 (883)	3,540 (1,407)	−7.4^*^	0.29	0.31^**^	3,094 (687)	3,290 (1,287)	6.4	0.37	0.40^**^	153
Proline, mg	4,467 (945)	4,636 (1,982)	3.8	0.28	0.30^**^	3,869 (748)	4,627 (1,953)	19.6^**^	0.27	0.29^**^	153
Serine, mg	4,095 (921)	4,073 (1,783)	−0.6	0.39	0.41^**^	3,475 (707)	3,961 (1,568)	14.0^**^	0.36	0.39^**^	153
Hydroxyproline, mg	306 (106)	252 (152)	−17.5^**^	0.21	0.24^*^	229 (86)	216 (130)	−5.8	0.19	0.22^*^	34

12d-WFR, 12-day weighed food record; CC, correlation coefficient; FFQ, food frequency questionnaire; SD, standard deviation.

^a^Percentage differences: (FFQ-12d-WFR)/12d-WFR × 100 (%). *P*-values refer to paired *t*-test between intakes by long-FFQ and those by 12d-WFR for each; ^*^P < 0.05, ^**^P < 0.01.

^b^Spearman’s rank correlation coefficients based on energy-adjusted values.

^c^Spearman’s rank correlation coefficients based on energy-adjusted values and expressed as deattenuated CC. ^*^P < 0.05, ^**^P < 0.01.

^d^Deattenuated CCx = energy-adjusted CCx × SQRT(1 + λx/n), where λx is the ratio of within- to between-individual variance for nutrient x and n is the number of dietary records (12 days).

^e^Number of items in long-FFQ covered by Food Composition Table 2020 in Japan.

**Table 2C.** Daily intakes of fatty acids according to the long-FFQ, and percentage differences between intakes by the long-FFQ and 12d-WFR and their correlations in men and women aged 40–74 years

	Men (*n* = 98)					Women (*n* = 142)					
	
	12d-WFR	Long-FFQ	%diff^a^	CC^b^	CC^c,d^	12d-WFR	Long-FFQ	%diff^a^	CC^b^	CC^c,d^	Number of items^e^
	
	Mean (SD)	Mean (SD)				Mean (SD)	Mean (SD)				
Butyric acid, mg	177 (127)	250 (279)	41.6^**^	0.46	0.51^**^	229 (163)	384 (401)	67.8^**^	0.43	0.47^**^	12
Hexanoic acid, mg	112 (81)	156 (178)	38.5^*^	0.46	0.51^**^	146 (104)	238 (253)	63.7^**^	0.43	0.46^**^	13
Heptanoic acid, mg	0.8 (0.8)	1.4 (2.4)	78.8^**^	0.48	0.51^**^	1.1 (1.0)	2.5 (3.3)	129.3^**^	0.47	0.50^**^	3
Octanoic acid, mg	81 (56)	96 (104)	18.5	0.45	0.51^**^	108 (72)	148 (151)	37.6^**^	0.39	0.43^**^	13
Decanoic acid, mg	170 (110)	223 (225)	31.4^*^	0.49	0.53^**^	213 (136)	331 (324)	55.2^**^	0.41	0.44^**^	32
Lauric acid, mg	332 (216)	318 (263)	−4.3	0.45	0.55^**^	420 (270)	465 (409)	10.9	0.40	0.47^**^	57
Tridecanoic acid, mg	2.4 (2.4)	4.3 (7.1)	77.5^**^	0.47	0.50^**^	3.3 (3.2)	7.4 (9.9)	121.1^**^	0.48	0.51^**^	3
Myristic acid, mg	1,248 (491)	1,375 (927)	10.2	0.38	0.43^**^	1,239 (535)	1,703 (1,287)	37.5^**^	0.37	0.41^**^	107
Pentadecanoic acid, mg	117 (49)	133 (97)	13.4	0.37	0.41^**^	117 (54)	165 (133)	40.8^**^	0.40	0.44^**^	81
Ant-pentadecanoic acid, mg	26.1 (18.7)	38.3 (41.8)	46.8^**^	0.45	0.49^**^	33.8 (24.1)	57.6 (59.9)	70.5^**^	0.43	0.46^**^	11
Palmitic acid, mg	10,531 (3,127)	10,310 (5,212)	−2.1	0.42	0.46^**^	9,089 (2,592)	10,713 (5,313)	17.9^**^	0.35	0.38^**^	156
Iso-palmitic acid, mg	12.4 (9.0)	17.8 (20.2)	43.9^**^	0.46	0.51^**^	16.1 (11.6)	27.0 (29.0)	68.0^**^	0.42	0.46^**^	10
Heptadecanoic acid, mg	163 (55)	162 (89)	−0.8	0.36	0.43^**^	134 (46)	162 (97)	20.9^**^	0.35	0.40^**^	82
Ant-heptadecanoic acid, mg	24.7 (17.7)	36.4 (39.7)	47.2^**^	0.46	0.50^**^	32.2 (22.7)	55.4 (56.7)	72.3^**^	0.42	0.45^**^	11
Stearic acid, mg	4,254 (1,444)	4,192 (2,217)	−1.5	0.41	0.46^**^	3,688 (1,208)	4,425 (2,325)	20.0^**^	0.43	0.47^**^	149
Arachidic acid, mg	174 (50)	180 (87)	3.4	0.33	0.37^**^	157 (47)	200 (102)	27.6^**^	0.19	0.21^*^	103
Behenic acid, mg	87.5 (31.1)	110.1 (79.0)	25.7^**^	0.26	0.32^*^	87.7 (40.2)	120.7 (100.9)	37.7^**^	0.19	0.25^*^	71
Lignoceric acid, mg	40.7 (14.3)	56.4 (39.3)	38.6^**^	0.25	0.32^*^	39.2 (18.5)	60.0 (51.7)	53.1^**^	0.27	0.34^**^	49
Decenoic acid, mg	14.5 (10.4)	21.0 (22.9)	44.8^**^	0.46	0.51^**^	18.5 (13.3)	31.4 (32.6)	69.4^**^	0.43	0.46^**^	13
Myristoleic acid, mg	89.9 (46.5)	122.1 (100.7)	35.8^**^	0.28	0.35^**^	89.8 (50.9)	129.5 (116.2)	44.2^**^	0.46	0.53^**^	35
Pentadecenoic acid, mg	0.2 (0.2)	0.1 (0.1)	−43.0^**^	0.15	0.27	0.1 (0.1)	0.1 (0.1)	−4.2	0.08	0.23	1
Palmitoleic acid, mg	1,062 (327)	993 (563)	−6.5	0.40	0.47^**^	825 (254)	898 (482)	9.0	0.29	0.34^**^	117
Heptadecenoic acid, mg	113 (39)	113 (68)	0.4	0.38	0.48^**^	88 (32)	102 (61)	15.4^**^	0.36	0.43^**^	62
Oleic acid, mg	7,408 (2,697)	6,909 (4,637)	−6.7	0.26	0.32^**^	5,805 (1,811)	6,257 (3,951)	7.8	0.15	0.19	31
Cis–vaccenic acid, mg	464 (173)	419 (266)	−9.6	0.23	0.28^*^	350 (120)	372 (209)	6.2	0.16	0.21	27
Icosenoic acid, mg	770 (358)	637 (366)	−17.2	0.21	0.35^*^	590 (274)	643 (377)	8.9	0.22	0.38^**^	98
Docosenoic acid, mg	528 (396)	398 (316)	−24.5^**^	0.19	0.35	395 (288)	400 (322)	1.1	0.25	0.57^**^	46
Tetracosenoic acid, mg	63.6 (30.6)	49.7 (30.9)	−21.8^**^	0.29	0.42^**^	46.7 (23.0)	50.7 (30.9)	8.6	0.26	0.39^**^	37
Hexadecadienoic acid, mg	15.5 (9.2)	12.1 (9.9)	−22.0^**^	0.29	0.46^**^	11.3 (7.9)	11.7 (10.0)	4.0	0.35	0.47^**^	11
Hexadecatrienoic acid, mg	12.5 (6.6)	10.6 (7.7)	−15.0^**^	0.28	0.39^**^	10.3 (6.0)	11.3 (8.5)	9.1	0.37	0.48^**^	15
Hexadecatetraenoic acid, mg	13.6 (8.9)	11.7 (9.7)	−14.5	0.26	0.42^*^	10.1 (7.2)	11.4 (9.6)	12.7	0.37	0.60^**^	10
Linoleic acid, mg	10,481 (2,905)	10,563 (5,406)	0.8	0.41	0.45^**^	9,326 (2,502)	10,792 (4,955)	15.7^**^	0.29	0.32^**^	155
α-linolenic acid, mg	1,568 (459)	1,584 (895)	1.0	0.31	0.35^**^	1,412 (422)	1,756 (885)	24.4^**^	0.28	0.31^**^	145
γ-linolenic acid, mg	5.4 (3.7)	5.6 (4.3)	2.4	0.31	0.45^**^	4.5 (3.0)	6.5 (5.0)	42.2^**^	0.13	0.20	14
Octadecatetraenoic acid, mg	127.7 (91.5)	89.1 (73.1)	−30.2^**^	0.22	0.38^*^	92.0 (67.5)	87.6 (74.0)	−4.8	0.24	0.48^**^	25
Icosadienoic acid, mg	77.9 (28.4)	66.2 (38.5)	−15.0^**^	0.33	0.39^**^	56.2 (20.7)	60.2 (31.2)	7.1	0.32	0.37^**^	55
Icosatrienoic acid (n-3), mg	4.0 (3.4)	1.7 (1.5)	−58.5^**^	0.10	0.20	3.0 (2.8)	1.7 (1.6)	−43.4^**^	0.18	0.35^*^	4
Icosatrienoic acid (n-6), mg	36.4 (11.8)	35.8 (24.7)	−1.4	0.43	0.49^**^	29.5 (9.0)	34.6 (19.9)	17.4^**^	0.33	0.37^**^	49
Icosatetraenoic acid (n-3), mg	48.1 (27.1)	35.9 (25.4)	−25.4^**^	0.22	0.31^*^	36.0 (21.0)	36.4 (26.9)	1.0	0.28	0.41^**^	25
Arachidonic acid, mg	194 (59)	170 (147)	−12.2	0.46	0.51^**^	151 (45)	157 (102)	3.5	0.35	0.40^**^	52
Icosapentaenoic acid, mg	445 (228)	311 (223)	−30.2^**^	0.27	0.36^**^	325 (179)	305 (226)	−6.0	0.35	0.47^**^	41
Henicosapentaenoic acid, mg	14.4 (8.9)	10.5 (8.5)	−27.5^*^	0.24	0.37^*^	10.9 (7.2)	10.3 (8.6)	−5.7	0.30	0.46^**^	12
Docosadienoic acid, mg	1.2 (0.8)	0.6 (0.5)	−47.7^**^	−0.02	−0.03	1.0 (0.7)	0.7 (0.5)	−32.0^**^	0.19	0.36^*^	5
Docosatetraenoic acid, mg	22.6 (7.9)	19.2 (14.0)	−15.1^*^	0.31	0.36^**^	16.7 (5.8)	16.5 (10.0)	−1.4	0.23	0.28^**^	23
Docosapentaenoic acid (n-3), mg	122.8 (59.7)	96.5 (63.2)	−21.4^**^	0.29	0.38^**^	89.6 (45.1)	94.2 (64.9)	5.2	0.32	0.42^**^	42
Docosapentaenoic acid (n-6), mg	34.8 (12.4)	30.6 (34.8)	−12.3	0.36	0.44^**^	27.7 (10.2)	27.0 (24.3)	−2.6	0.33	0.40^**^	23
Docosahexaenoic acid, mg	755 (368)	536 (361)	−29.1^**^	0.27	0.37^**^	549 (281)	520 (368)	−5.2	0.31	0.43^**^	33

12d-WFR, 12-day weighed food record; CC, correlation coefficient; FFQ, food frequency questionnaire; SD, standard deviation.

^a^Percentage differences: (FFQ-12d-WFR)/12d-WFR × 100 (%). *P*-values refer to paired *t*-test between intakes by long-FFQ and those by 12d-WFR for each; ^*^P < 0.05, ^**^P < 0.01.

^b^Spearman’s rank correlation coefficients based on energy-adjusted values.

^c^Spearman’s rank correlation coefficients based on energy-adjusted values and expressed as deattenuated CC. ^*^P < 0.05, ^**^P < 0.01.

^d^Deattenuated CCx = energy-adjusted CCx × SQRT(1 + λx/n), where λx is the ratio of within- to between-individual variance for nutrient x and n is the number of dietary records (12 days).

^e^Number of items in long-FFQ covered by Food Composition Table 2020 in Japan.

The deattenuated CCs of each nutrient with the long-FFQ were slightly lower in women than in men. The median values of deattenuated CCs of energy-adjusted nutrient intakes based on the long-FFQ in men and women were 0.52 (range, −0.08 for mannitol to 0.72 for available carbohydrates; monosaccharide equivalents and mass matter and sorbitol) and 0.42 (range, −0.09 for low-molecular-weight water-soluble dietary fiber to 0.77 for galactose) for sugars, 0.38 (range, 0.24 for hydroxyproline to 0.46 for aspartic acid) and 0.37 (range, 0.22 for hydroxyproline to 0.49 for arginine and aspartic acid) for amino acids, and 0.42 (range, −0.03 for docosadienoic acid to 0.55 for lauric acid) and 0.42 (range, 0.19 for Oleic acid to 0.60 for hexadecatetraenoic acid) for fatty acids, respectively (Table 2A, Table 2B, and Table 2C). CCs of available carbohydrate intake from the long-FFQ were reasonable regardless of whether calculation was by monosaccharide equivalents, mass matter or calculated by differences in both men and women. CCs of amino acid intakes from the long-FFQ were also good or acceptable in both men and women. For fatty acid intake, differences between men and women were seen in the CCs of oleic acid, γ-linolenic acid, icosatrienoic acid (n-3) and docosadienoic acid. Although almost all nutrient intakes based on the short-FFQ were underestimated compared with those based on the long-FFQ in men and women, the CCs of these nutrient intakes between the 12-day WFR and short-FFQ were similar to those for the long-FFQ in both men and women. The median values and deattenuated CCs of intakes of energy-adjusted sugar, amino acids, and fatty acids based on the short-FFQ in men and women were 0.46 (range, −0.19 for succinic acid to 0.69 for available carbohydrates; monosaccharide equivalents) in men and 0.38 (range, −0.16 for low-molecular-weight water-soluble dietary fiber to 0.75 for lactose) in women for sugar; 0.33 (range, 0.21 for methionine to 0.39 for serine) and 0.45 (range, 0.38 for hydroxyproline to 0.54 for aspartic acid) for amino acids; and 0.42 (range, 0.07 for docosadienoic acid to 0.58 for heptanoic acid and tridecanoic acid) and 0.43 (range, 0.10 for behenic acid to 0.70 for tridecanoic acid) for fatty acids, respectively (eTable 1A, eTable 1B, and eTable 1C).

eTable 2 and eTable 3 show percentage differences and correlations of intake of energy and 54 nutrients as assessed by 12-day WFR and long- and short-FFQs for men and women, respectively. Validation of intakes of energy and major nutrients estimated from the long- and short-FFQ were good or acceptable for many nutrients, and similar to those in the previous study.^12^ ICC for reproducibility ranged from 0.23 to 0.77 (median, 0.52) in men and 0.37 to 0.74 (median, 0.53) in women for sugars; 0.54 to 0.63 (median, 0.58) in men and 0.40 to 0.58 (median, 0.46) in women for amino acids; 0.20 to 0.61 (median, 0.54) in men and 0.31 to 0.61 (median, 0.53) in women for fatty acids; and 0.27 to 0.90 (median, 0.57) in men and 0.39 to 0.85 (median, 0.52) in women for energy and major nutrients (eTable 4).

## DISCUSSION

We evaluated the validity of estimated intakes of sugars, amino acids, and fatty acids from a long-FFQ in middle-aged Japanese men and women using a 12-day WFR as reference method. The deattenuated CCs for energy-adjusted intakes of sugars, amino acids, and fatty acids between the long-FFQ and 12-day WFR were good or acceptable for most nutrients. Although almost all nutrient intakes based on the short-FFQ were underestimated compared with those based on the long-FFQ, the CCs of the short-FFQ were similar to those for the long-FFQ. CCs of most nutrients were reasonable, although some nutrients showed different trends in men and women, and their use in epidemiological studies requires caution.

We conducted additional analysis of participants stratified by age group. The middle-aged group (40–64 years) consisted of 78 men and 113 women and the elderly group (65–74 years) of 20 men and 29 women. The deattenuated CC of sugar was better in the middle-aged group than in the elderly group for both men and women. The deattenuated CC of amino acids was better in the middle-aged group than in the elderly group in women. On the other hand, in men, the deattenuated CC of fatty acids was better in the elderly group than in the middle-aged group. In men, the median values of deattenuated CCs of energy-adjusted nutrient intakes based on the long-FFQ were 0.52 (range, −0.16 for mannitol to 0.72 for available carbohydrates; monosaccharide equivalents and mass matter and sucrose) for sugars, 0.37 (range, 0.25 for hydroxyproline to 0.42 for aspartic acid) for amino acids, and 0.42 (range, −0.02 for docosadienoic acid to 0.55 for lauric acid) for fatty acids in the middle-aged group; and 0.47 (range, −0.08 for oxalic acid to 2.00 for tartaric acid), 0.32 (range, 0.12 for hydroxyproline to 0.49 for aspartic acid), and 0.50 (range, −0.15 for docosadienoic acid to 1.41 for docosenoic acid) in the elderly group. In women, these values were 0.46 (range, −0.03 for low-molecular-weight water-soluble dietary fiber to 0.76 for water-insoluble dietary fiber) for sugars, 0.29 (range, 0.21 for hydroxyproline to 0.41 for aspartic acid) for amino acids, and 0.38 (range, 0.04 for pentadecenoic acid to 0.55 for myristoleic and stearic acid) for fatty acids in the middle-aged group; and 0.25 (range, −0.34 for low-molecular-weight water-soluble dietary fiber to 0.88 for succinic acid), 0.42 (range, 0.20 for hydroxyproline to 0.61 for sulfur-containing amino acids), and 0.36 (range, −0.03 for behenic acid to 2.60 for pentadecenoic acid) in the elderly group. Because of the small number of elderly participants in both men and women, deattenuated CCs could not be calculated for some nutrients (data not shown).

Validity for available carbohydrates was reasonable no matter whether the calculation was by monosaccharide equivalents, mass matter or calculated by differences. This was likely because we substituted the value of available carbohydrate calculation by differences when the value of available carbohydrates expressed in monosaccharide equivalents or mass matter in foods was missing. This in turn indicates that use of any available carbohydrate value is possible, depending on purpose. The validation of available carbohydrates (monosaccharides [glucose, fructose, and galactose], disaccharides [sucrose, maltose, and lactose], and polysaccharides [starch]) showed slightly better CCs than those from the former JPHC study^14^; that study used FCT 2015, which measured 75 of 147 food items (51%) in the FFQ, with the remaining items being substituted.^14^ In the present study using FCT 2020, sugar measurements were increased to 102 of 172 food and beverage items and 11 seasonings in the long-FFQ (56%).

In the previous study, water-soluble and -insoluble dietary fiber were based on FCT 2010, in which total, water-soluble, and -insoluble dietary fiber were measured using the conventional method, namely the Prosky or modified Prosky methods (in turn based on AOAC.985.29 method). In contrast, in FCT 2020, dietary fiber was additionally measured by the AOAC.2011.25 method, which is also recommended by the Codex Alimentarius Commission. The AOAC.2011.25 method additionally quantifies low-molecular-weight water-soluble dietary fiber, such as oligosaccharides and indigestible starch, and these values were accordingly included in total dietary fiber in FCT 2020. Thus, calculation of water-soluble dietary fiber using the AOAC 2011.25 method is more comprehensive than that using the conventional method. As examples, water-soluble dietary fiber is 0.9 g/100 g by the conventional method versus 2.2 g/100 g by the AOAC.2011.25 method in boiled soybeans; 0.4 g/100 g and 1.9 g/100 g in plain bread; and 0 g/100 g and 0.9 g/100 g in white rice (polished rice). For this reason, total dietary fiber is also higher with the AOAC.2011.25 method than with the conventional method. To allow comparison with the previous study,^12^ total and water-soluble and -insoluble dietary fiber using the conventional method are included in eTable 2 and eTable 3. The validation of total, water-soluble, and -insoluble dietary fiber estimates using conventional methods was similar to those in the previous study.^12^ In the present study, dietary fiber newly measured using the AOAC.2011.25 method is included in Table 2A. Validity of total dietary fiber as a combination of both the conventional and AOAC.2011.25 methods was reasonable. In contrast, CCs of total, low-molecular-weight water-soluble, and water-insoluble dietary fiber measured using only the AOAC.2011.25 method were low, likely because of the small number of measurement items in FCT 2020, and not all long-FFQ items were measured. The median percentage of measurement items of 54 major nutrients in the long-FFQ was 73.2%, whereas that of total, water-soluble, and -insoluble dietary fiber measured using the AOAC.2011.25 method was only 5.5%. Confirmation of the validity of these dietary fiber values requires measurement of a greater number of food items using the AOAC.2011.25 method.

CCs of amino acid intake from the long-FFQ were good or acceptable in both men and women, albeit slightly better in men than in women. Results were slightly better than those in the former JPHC study,^13^ which used FCT 2010 and measured only 337 amino acids items, with the remaining items substituted from another database in Japan.^13^ As amino acids were measured in 1,951 items (79% of total food items) with FCT 2020, we considered that the measured intakes of amino acids in this validation study were likely more accurate.

This study also examined the validity of specific items among fatty acids. CCs of some fatty acids were low, namely icosatrienoic acid (n-3) and docosadienoic acid for men. It is considered that the content of these nutrients in each food was low or was measured in only a small number of food items. The number of food items measured for fatty acids with the FCT increased from 471 items in 1989 to 1919 (77% of total food items) in 2020. A previous study^12^ examined major fatty acids, such as polyunsaturated fatty acids or saturated fatty acid, using FCT2005, but not the less common fatty acids. Recently, some of these less common fatty acids, such as arachidonic acid, have been examined for their effect on cardiovascular diseases^23^ and cancer.^24^ Our present results may, therefore, be beneficial for future epidemiological studies in Japanese subjects.

The validity of the estimated intakes of energy and 54 major nutrients was reasonable, apart from iodine in both men and women (eTable 2 and eTable 3), and results were similar to those of the previous study.^12^ Energy intake was slightly lower using FCT 2020 than FCT 2010, mainly because energy was calculated primarily from carbohydrate, protein, and fat in FCT 2010 but in FCT 2020 from the amount of protein using amino acid composition, which has lower values than protein; the triacylglycerol equivalent of fatty acids, which has lower values than fat; and available carbohydrates (3.75 kcal/g), whose energy conversion factor is lower than that of carbohydrates (4 kcal/g).

To calculate the deattenuated CCs of habitual nutrient intake between the WFR and FFQ in the present study, energy adjustment was conducted after calculating the average over the 12 days of WFRs. In addition, the deattenuated value was corrected using the ratios of the intra- to inter-individual variances based on the crude intake of individual nutrients in the 12-day WFR. However, another method of calculating the deattenuated CC involves use of the mean nutrient intake for WFRs after energy adjustment by day, together with the ratios of the intra- to inter-individual variances based on the energy adjusted intake of individual nutrients in the 12-day WFR. Results for the deattenuated CCs using this second method were similar to those with the first method (data not shown).

### Strengths and limitations

Although caution is warranted for some nutrients, dietary intakes of sugars, amino acids, and fatty acids estimated using the long-FFQ showed acceptable relative validity. In addition, energy intake was estimated using measurement methods recommended by the Food and Agriculture Organization, which likely provided more accurate values.

Nevertheless, our study also had a number of limitations. First, although we examined the validity of estimated intakes of sugars, amino acids, and fatty acids, some nutrients had large over- or underestimation, despite the increase in measurement items. It was considered that there are some nutrients with low intakes or few measured items in the food composition table. Some nutrients, such as gluconic acid, succinic acid, fumaric acid, tartaric acid, chlorogenic acid, and pantadecenoic acid, are measured in only a few food items in FCT 2020 and are also found in only a few applicable items in the long-FFQ (eg, 1 or 2). Intakes of these nutrients estimated from the WFR or FFQ were also low (about 0.1 mg/day to 1 mg/day for both men and women) (Table 1). In the case of participants who did not take these nutrients even in the 12-day WFR, these nutrients have large intra- and inter-individual variation, and this should be considered when interpreting these results, even allowing that we calculated deattenuated CCs. Caution is also required in the use of individual nutrients in epidemiological studies. Second, even using the 12-day WFR collected in four seasons and over 200 samples, this sampling may not be sufficiently long enough to act as a gold standard for nutrients with large intra-individual variation. We could not find reported intra- and inter-individual variation for nutrients, such as sugar or amino acids, and the number of days necessary to evaluate the habitual intake of these nutrients is unknown. Regarding fatty acids, the estimated number of days needed is more than 12 days for main fatty acids, such as poly-unsaturated fatty acid or n-3 fatty acid.^25^^,^^26^ In addition, intra- and inter-individual variation was also considered to differ by age and sex.^26^ Moreover, as mentioned above, some nutrients occurred in only a few food items and were subject to large variation. Accordingly, the deattenuated CCs may have been over- or underestimated for some nutrients. However, no studies have examined the validity of these nutrients, and further studies are needed to confirm our results. Third, some nutrients of sugars, amino acids, or fatty acids may be taken as supplements or fortified foods, albeit that this would not have influenced the validity of estimated intake using the FFQ because the reference method—intake using the WFR—also did not include such foods due to the lack of a database for dietary supplements. Moreover, the percentage of users of supplements other than vitamin C and E or multivitamins was low (about 7.8% from WFR) in this population.^27^

In conclusion, compared with the 12-day WFR, the long-FFQ used in the JPHC-NEXT study provided acceptable relative validity of most nutrient intakes of sugars, amino acids, and fatty acids in the Japanese men and women of this study population. Regarding dietary fiber, the use of dietary fiber intake measured only by the AOAC.2011.25 method for epidemiological studies hampers accurate evaluation because it has been applied to measurement in only a few food items. When comparing total dietary fiber intake with the previous value, it is better to use the value measured using the conventional method (Prosky or modified Prosky method). When not comparing with previous intake, it is preferable to use total dietary fiber as the combined intake measured using the conventional and the AOAC.2011.25 methods. Although caution is warranted for some nutrients, these findings may be useful in assessing the association of intakes of sugars, amino acids, and fatty acids with health conditions, such as chronic diseases, in Japanese subjects.

## SUPPLEMENTARY MATERIAL

eTable 1A. Daily intakes of sugars according to the short-FFQ, and percentage differences between intakes by the short-FFQ and 12d-WFR and their correlations in men and women aged 40–74 years

eTable 1B. Daily intakes of amino acids according to the short-FFQ, and percentage differences between intakes by the short-FFQ and 12d-WFR and their correlations in men and women aged 40–74 years

eTable 1C. Daily intakes of fatty acids according to the short-FFQ, and percentage differences between intakes by the short-FFQ and 12d-WFR and their correlations in men and women aged 40–74 years

eTable 2. Daily intakes of energy and major nutrients according to the long-FFQ, and percentage differences between intakes by the long-FFQ and 12d-WFR and their correlations in men and women aged 40–74 years

eTable 3. Daily intakes of energy and major nutrients according to the short-FFQ, and percentage differences between intakes by the short-FFQ and 12d-WFR and their correlations in men and women aged 40–74 years

eTable 4. Daily intakes of energy and nutrients according to the FFQs and their intra-correlations among men and women aged 40–74 years
