# Supplementary material for: Validity of the Intake of Sugars, Amino Acids, and Fatty Acids Estimated Using a Self-administered Food Frequency Questionnaire in Middle-aged and Elderly Japanese: The Japan Public Health Center-based Prospective Study for the Next Generation (JPHC-NEXT) Protocol Area
Source: J Epidemiol. 2024 Aug 5;34(8):372–9. doi: 10.2188/jea.JE20230132 (PMC11230880; doi:10.2188/jea.JE20230132)
Supplement: Supplementary file 1 [file je-34-372-s001.pdf]

eTable 1A. Daily intakes of sugars according to the short-FFQ, and percentage differences between intakes by the short-FFQ and 12d-WFR and their correlations in men and women aged 40–74 years

	Men (n=92)					Women (n=136)				
	12d-WFR Mean (SD)	Short-FFQ Mean (SD)	%diff [a]	CC [b]	CC [c, d]	12d-WFR Mean (SD)	Short-FFQ Mean (SD)	%diff [a]	CC [b]	CC [c, d]
Available carbohydrate; monosaccharide equivalents, g	305.0 (63.3)	251.4 (76.8)	-17.6**	0.66	0.69**	248.2 (38.6)	204.1 (51.9)	-17.8**	0.41	0.44**
Available carbohydrate; mass matter, g	281.7 (58.3)	230.9 (70.3)	-18.0**	0.65	0.68**	230.0 (36.0)	187.9 (48.0)	-18.3**	0.41	0.43**
Available carbohydrate; calculated by difference, g	287.8 (59.8)	238.0 (72.5)	-17.3**	0.65	0.68**	235.0 (35.8)	193.9 (49.3)	-17.5**	0.40	0.43**
Starch, g	187.8 (48.1)	172.9 (63.7)	-7.9*	0.55	0.58**	137.3 (27.4)	131.8 (33.8)	-4.0	0.47	0.50**
Glucose, g	11.9 (5.0)	5.4 (4.1)	-54.5**	0.55	0.58**	11.0 (4.0)	5.0 (3.1)	-54.9**	0.32	0.35**
Fructose, g	9.2 (4.5)	5.1 (3.7)	-44.3**	0.51	0.54**	10.3 (4.4)	7.2 (4.5)	-30.2**	0.46	0.50**
Sucrose, g	22.3 (11.7)	8.8 (7.2)	-60.5**	0.56	0.56**	28.0 (9.9)	12.9 (9.8)	-54.0**	0.49	0.55**
Maltose, g	0.9 (0.5)	0.5 (0.3)	-47.6**	0.37	0.46**	1.1 (0.5)	0.6 (0.6)	-44.2**	0.42	0.67**
Lactose, g	4.5 (3.6)	4.5 (7.1)	0.2	0.59	0.62**	6.2 (4.4)	5.1 (5.3)	-17.8*	0.72	0.75**
Trehalose, g	0.3 (0.2)	0.1 (0.2)	-50.6**	0.13	0.18	0.2 (0.1)	0.2 (0.2)	-26.0**	0.13	0.19
Total dietary fiber [e], g	24.5 (6.9)	10.2 (5.0)	-58.3**	0.52	0.54**	22.1 (5.8)	11.8 (5.4)	-46.6**	0.48	0.50**
Total dietary fiber (AOAC.2011.25) [f], g	11.0 (3.5)	2.6 (1.9)	-76.1**	0.05	0.05	8.1 (2.2)	2.3 (2.1)	-71.6**	-0.08	-0.10
Low-molecular-weight water-soluble dietary fiber [g], g	4.6 (1.6)	0.9 (0.7)	-80.5**	-0.02	-0.02	3.2 (0.9)	0.7 (0.6)	-76.5**	-0.15	-0.16
High-molecular-weight water-soluble dietary fiber [h], g	1.7 (0.9)	0.6 (0.5)	-63.3**	0.23	0.28*	1.4 (0.6)	0.6 (0.5)	-59.1**	0.06	0.08
Water-insoluble dietary fiber [i], g	4.7 (1.6)	1.0 (0.7)	-77.7**	0.09	0.10	3.6 (1.1)	1.0 (1.1)	-72.9**	-0.05	-0.07
Resistant starch, g	0.7 (0.3)	0.3 (0.2)	-56.8**	0.43	0.49**	0.5 (0.2)	0.3 (0.5)	-35.9**	0.28	0.33**
Sorbitol, g	0.3 (0.3)	0.2 (0.2)	-39.2**	0.49	0.64**	0.4 (0.4)	0.3 (0.2)	-37.2**	0.41	0.49**
Mannitol, g	0.17 (0.21)	0.01 (0.01)	-95.3**	-0.10	-0.15	0.16 (0.19)	0.01 (0.01)	-93.7**	0.04	0.06
Acetic acid, g	0.258 (0.214)	0.008 (0.013)	-96.8**	0.14	0.16	0.230 (0.146)	0.008 (0.014)	-96.3**	0.07	0.08
Lactic acid, g	0.498 (0.425)	0.010 (0.044)	-97.9**	0.003	0.003	0.532 (0.343)	0.005 (0.018)	-99.1**	-0.04	-0.05
Oxalic acid, g	0.10 (0.08)	0.08 (0.18)	-14.1	0.34	0.51**	0.10 (0.10)	0.11 (0.13)	11.2	0.28	0.39**
Succinic acid, g	0.018 (0.067)	0.010 (0.044)	-43.6	-0.17	-0.19	0.007 (0.020)	0.005 (0.018)	-28.4	0.24	0.33**
Fumaric acid, g	0.001 (0.002)	0.005 (0.011)	590.6**	0.05	0.06	0.001 (0.001)	0.010 (0.010)	787.6**	0.07	0.14
Malic acid, g	0.5 (0.3)	0.3 (0.3)	-46.1**	0.53	0.59**	0.5 (0.3)	0.4 (0.2)	-32.6**	0.42	0.46**
Tartaric acid, g	0.04 (0.08)	0.01 (0.06)	-61.9**	-0.08	-0.09	0.03 (0.04)	0.01 (0.02)	-75.2**	0.15	0.26
Citric acid, g	0.8 (0.4)	0.5 (0.4)	-41.2**	0.37	0.42**	0.9 (0.4)	0.6 (0.3)	-41.2**	0.49	0.54**
Ferulic acid, mg	1.4 (1.4)	1.2 (2.2)	-9.5	0.29	0.36**	1.5 (1.2)	1.8 (1.8)	17.4	0.26	0.38**

12d-WFR, 12-day weighed food record; CC, correlation coefficient; FFQ, food frequency questionnaire; SD, standard deviation.

[a] Percentage differences: (FFQ-12d-WFR)/12d-WFR × 100 (%). *P*-values refer to the paired t-test between intakes by short-FFQ and those by 12d-WFR for each; * *P*<0.05, ** *P*<0.01.

[b] Spearman's rank correlation coefficients based on energy-adjusted values.

[c] Spearman's rank correlation coefficients based on energy-adjusted values and expressed as deattenuated CC. * *P*<0.05, ** *P*<0.01.

[d] Deattenuated CCx = energy-adjusted CCx × SQRT(1 + λx/n), where λx is the ratio of within- to between-individual variance for nutrient x and n is the number of dietary records (12 days).

[e] Total dietary fiber was derived by combination of the AOAC.2011.25 method with either the Proskey or modified Prosky method.

[f] Total dietary fiber (AOAC.2011.25) measured using only the AOAC.2011.25 method.

[g] Low-molecular-weight water-soluble dietary fiber that remains soluble in 78% aqueous ethanol.

[h] High-molecular-weight water-soluble dietary fiber that precipitates from 78% aqueous ethanol.

[i] Water-insoluble dietary fiber measured using only the AOAC.2011.25 method.

eTable 1B. Daily intakes of amino acids according to the short-FFQ, and percentage differences between intakes by the short-FFQ and 12d-WFR and their correlations in men and women aged 40–74 years

	Men (n=92)					Women (n=136)				
	12d-WFR	Short-FFQ	%diff [a]	CC [b]	CC [c, d]	12d-WFR	Short-FFQ	%diff [a]	CC [b]	CC [c, d]
	Mean (SD)	Mean (SD)				Mean (SD)	Mean (SD)			
Isoleucine, mg	3,524 (791)	2,643 (1,131)	-25.0**	0.33	0.35**	2,941 (624)	2,299 (840)	-21.8**	0.43	0.45**
Leucine, mg	6,219 (1,363)	4,734 (1,958)	-23.9**	0.30	0.32**	5,188 (1,073)	4,110 (1,469)	-20.8**	0.41	0.44**
Lysine, mg	5,336 (1,316)	3,851 (1,842)	-27.8**	0.29	0.31**	4,402 (1,050)	3,405 (1,412)	-22.6**	0.40	0.43**
Methionine, mg	1,865 (424)	1,408 (596)	-24.5**	0.20	0.21	1,511 (331)	1,209 (455)	-20.0**	0.39	0.42**
Cystine, mg	1,208 (245)	986 (372)	-18.4**	0.32	0.34**	992 (181)	829 (255)	-16.4**	0.42	0.45**
Sulfur-containing amino acids, mg	3,089 (665)	2,392 (957)	-22.6**	0.23	0.25*	2,513 (508)	2,035 (700)	-19.0**	0.39	0.42**
Phenylalanine, mg	3,670 (779)	2,813 (1,133)	-23.3**	0.34	0.35**	3,071 (608)	2,434 (833)	-20.7**	0.45	0.48**
Tyrosine, mg	2,945 (650)	2,269 (951)	-22.9**	0.36	0.38**	2,455 (514)	1,955 (692)	-20.4**	0.42	0.45**
Aromatic amino acids, mg	6,631 (1,432)	5,108 (2,101)	-23.0**	0.34	0.36**	5,540 (1,127)	4,406 (1,531)	-20.5**	0.43	0.46**
Threonine, mg	3,473 (795)	2,569 (1,118)	-26.0**	0.29	0.31**	2,866 (625)	2,230 (834)	-22.2**	0.42	0.45**
Tryptophan, mg	991 (218)	761 (316)	-23.2**	0.33	0.35**	825 (171)	658 (230)	-20.2**	0.43	0.46**
Valine, mg	4,230 (926)	3,219 (1,314)	-23.9**	0.30	0.32**	3,521 (729)	2,780 (977)	-21.0**	0.41	0.44**
Histidine, mg	2,648 (634)	2,062 (941)	-22.1**	0.22	0.24*	2,111 (488)	1,798 (719)	-14.8**	0.41	0.45**
Arginine, mg	5,129 (1,152)	3,879 (1,590)	-24.4**	0.27	0.29**	4,167 (916)	3,297 (1,145)	-20.9**	0.47	0.50**
Alanine, mg	4,217 (966)	3,163 (1,302)	-25.0**	0.23	0.25*	3,398 (753)	2,711 (985)	-20.2**	0.44	0.47**
Aspartic acid, mg	7,662 (1,791)	5,688 (2,459)	-25.8**	0.34	0.36**	6,397 (1,442)	4,956 (1,807)	-22.5**	0.51	0.54**
Glutamic acid, mg	15,238 (3.088)	10,752 (4,165)	-29.4**	0.35	0.37**	12,859 (2,371)	9,564 (3,326)	-25.6**	0.44	0.47**
Glycine, mg	3,872 (864)	2,790 (1,148)	-28.0**	0.22	0.24*	3,101 (680)	2,411 (883)	-22.2**	0.42	0.45**
Proline, mg	4,530 (916)	3,305 (1,309)	-27.0**	0.33	0.35**	3,872 (750)	2,923 (1,082)	-24.5**	0.44	0.48**
Serine, mg	4,154 (896)	3,165 (1,347)	-23.8**	0.37	0.39**	3,486 (704)	2,710 (946)	-22.3**	0.45	0.47**
Hydroxyproline, mg	310 (105)	198 (130)	-36.3**	0.29	0.33**	230 (86)	173 (106)	-24.8**	0.32	0.38**

12d-WFR, 12-day weighed food record; CC, correlation coefficient; FFQ, food frequency questionnaire; SD, standard deviation.

[a] Percentage differences: (FFQ-12d-WFR)/12d-WFR × 100 (%).*P*-values refer to paired t-test between intakes by short-FFQ and those by 12d-WFR for each; * *P*<0.05, ** *P*<0.01.

[b] Spearman's rank correlation coefficients based on energy-adjusted values.

[c] Spearman's rank correlation coefficients based on energy-adjusted values and expressed as deattenuated CC. * *P*<0.05, ** *P*<0.01.

[d] Deattenuated CCx = energy-adjusted CCx × SQRT(1 + λx/n), where λx is the ratio of within- to between-individual variance for nutrient x and n is the number of dietary records (12 days).

eTable 1C. Daily intakes of fatty acids according to the short-FFQ, and percentage differences between intakes by the short-FFQ and 12d-WFR and their correlations in men and women aged 40–74 years

	Men (n=92)					Women (n=136)				
	12d-WFR Mean (SD)	Short-FFQ Mean (SD)	%diff [a]	CC [b]	CC [c, d]	12d-WFR Mean (SD)	Short-FFQ Mean (SD)	%diff [a]	CC [b]	CC [c, d]
Butyric acid, mg	179 (127)	113 (192)	-37.3**	0.43	0.48**	230 (165)	118 (141)	-48.7**	0.57	0.61**
Hexanoic acid, mg	114 (82)	74 (126)	-35.4**	0.45	0.49**	146 (106)	78 (93)	-47.0**	0.56	0.61**
Heptanoic acid, mg	0.8 (0.8)	0.9 (1.6)	8.9	0.55	0.58**	1.1 (1.0)	0.9 (1.2)	-13.7	0.64	0.68**
Octanoic acid, mg	82.9 (56.1)	43.0 (73.4)	-48.1**	0.41	0.46**	108.5 (72.9)	45.0 (53.9)	-58.5**	0.47	0.52**
Decanoic acid, mg	173 (110)	110 (159)	-36.1**	0.47	0.52**	214 (138)	113 (118)	-47.3**	0.56	0.60**
Lauric acid, mg	341 (217)	158 (181)	-53.7**	0.34	0.42**	424 (274)	160 (137)	-62.3**	0.40	0.46**
Tridecanoic acid, mg	2.5 (2.4)	2.7 (4.8)	8.9	0.54	0.58**	3.4 (3.3)	2.8 (3.6)	-16.5	0.66	0.70**
Myristic acid, mg	1,273 (482)	894 (705)	-29.8**	0.38	0.44**	1,244 (541)	852 (581)	-31.6**	0.48	0.52**
Pentadecanoic acid, mg	120 (48)	83 (72)	-30.7**	0.38	0.43**	118 (55)	79 (60)	-32.5**	0.54	0.59**
Ant-pentadecanoic acid, mg	26.5 (18.7)	17.4 (28.7)	-34.2**	0.42	0.46**	33.9 (24.4)	17.9 (21.1)	-47.1**	0.58	0.62**
Palmitic acid, mg	10,749 (3,058)	7,529 (4,101)	-30.0**	0.45	0.50**	9,100 (2,646)	6,490 (3,199)	-28.7**	0.32	0.35**
Iso-palmitic acid, mg	12.5 (9.0)	8.7 (14.3)	-30.9**	0.45	0.49**	16.1 (11.8)	8.9 (10.6)	-45.0**	0.58	0.62**
Heptadecanoic acid, mg	167 (54)	124 (81)	-25.3**	0.32	0.39**	134 (47)	107 (71)	-20.3**	0.39	0.44**
Ant-heptadecanoic acid, mg	25.1 (17.8)	16.1 (27.1)	-35.8**	0.44	0.48**	32.3 (23.0)	16.9 (19.9)	-47.6**	0.59	0.63**
Stearic acid, mg	4,345 (1426)	3,063 (1,881)	-29.5**	0.38	0.43**	3,692 (1,230)	2,584 (1,424)	-30.0**	0.36	0.39**
Arachidic acid, mg	178 (48)	107 (59)	-39.7**	0.31	0.35**	157 (48)	100 (44)	-36.4**	0.14	0.15
Behenic acid, mg	90.0 (30.2)	48.0 (31.4)	-46.7**	0.21	0.28*	88.5 (40.8)	45.5 (21.6)	-48.5**	0.07	0.10
Lignoceric acid, mg	41.7 (14.1)	23.3 (10.3)	-44.0**	0.34	0.44**	39.5 (18.8)	21.9 (9.3)	-44.6**	0.21	0.26*
Decenoic acid, mg	14.7 (10.4)	9.9 (16.0)	-32.8**	0.46	0.50**	18.6 (13.5)	10.3 (11.7)	-44.9**	0.59	0.64**
Myristoleic acid, mg	91.9 (46.4)	77.7 (74.8)	-15.5	0.22	0.28*	90.3 (51.6)	69.4 (72.1)	-23.2**	0.54	0.61**
Pentadecenoic acid, mg	0.2 (0.2)	0.1 (0.1)	-56.2**	0.12	0.23	0.1 (0.1)	0.1 (0.1)	-13.3	0.16	0.42
Palmitoleic acid, mg	1,084 (321)	765 (468)	-29.4**	0.45	0.53**	827 (258)	656 (420)	-20.6**	0.35	0.40**
Heptadecenoic acid, mg	115 (39)	83 (60)	-27.6**	0.28	0.36**	88 (33)	70 (56)	-20.9**	0.37	0.44**
Oleic acid, mg	7,554 (2,701)	5,425 (3,914)	-28.2**	0.33	0.40**	5,803 (1,836)	4,287 (2,800)	-26.1**	0.13	0.17
Cis–vaccenic acid, mg	471 (175)	346 (238)	-26.6**	0.34	0.43**	350 (123)	282 (189)	-19.3**	0.11	0.14
Icosenoic acid, mg	788 (359)	495 (307)	-37.2**	0.22	0.38*	595 (277)	488 (301)	-18.0**	0.23	0.38**
Docosenoic acid, mg	542 (402)	338 (261)	-37.6**	0.13	0.26	400 (290)	359 (269)	-10.3	0.23	0.52**
Tetracosenoic acid, mg	65.0 (30.8)	39.4 (26.3)	-39.4**	0.23	0.34*	47.1 (23.1)	39.4 (26.6)	-16.3**	0.23	0.34**
Hexadecadienoic acid, mg	15.6 (9.2)	10.3 (8.2)	-34.2**	0.26	0.43*	11.3 (7.9)	10.6 (8.2)	-6.3	0.31	0.41**
Hexadecatrienoic acid, mg	12.6 (6.5)	9.1 (7.0)	-28.3**	0.21	0.28*	10.4 (6.0)	10.1 (7.0)	-2.7	0.27	0.35**
Hexadecatetraenoic acid, mg	13.8 (8.9)	9.9 (8.1)	-28.6**	0.26	0.42*	10.1 (7.2)	10.2 (8.0)	1.0	0.32	0.52**
Linoleic acid, mg	10,720 (2,813)	7,207 (4,189)	-32.8**	0.43	0.48**	9,360 (2,545)	6,511 (2,919)	-30.4**	0.22	0.24*
α-linolenic acid, mg	1,605 (444)	1,042 (664)	-35.0**	0.32	0.38**	1,420 (428)	1,009 (484)	-29.0**	0.12	0.14
γ-linolenic acid, mg	5.5 (3.7)	3.1 (2.7)	-44.1**	0.33	0.46**	4.6 (3.0)	3.4 (2.8)	-25.9**	0.20	0.32*
Octadecatetraenoic acid, mg	130.9 (92.5)	76.3 (60.8)	-41.7**	0.15	0.28	92.9 (67.9)	80.2 (62.8)	-13.7	0.22	0.43**
Icosadienoic acid, mg	79.5 (28.5)	54.3 (36.5)	-31.7**	0.37	0.44**	56.3 (21.1)	44.5 (26.3)	-21.0**	0.29	0.34**
Icosatrienoic acid (n-3), mg	4.1 (3.5)	1.4 (1.2)	-66.6**	0.12	0.23	3.0 (2.8)	1.5 (1.3)	-49.2**	0.21	0.41*
Icosatrienoic acid (n-6), mg	37.1 (11.7)	29.1 (18.9)	-21.6**	0.47	0.53**	29.6 (9.1)	23.5 (13.9)	-20.6**	0.41	0.46**
Icosatetraenoic acid (n-3), mg	49.0 (27.5)	32.0 (22.8)	-34.7**	0.19	0.28	36.4 (21.2)	32.6 (22.6)	-10.4	0.30	0.45**
Arachidonic acid, mg	197 (58)	148 (126)	-25.2**	0.34	0.38**	152 (45)	112 (58)	-26.1**	0.28	0.32**
Icosapentaenoic acid, mg	453 (229)	271 (198)	-40.1**	0.27	0.37**	327 (180)	274 (195)	-16.4**	0.34	0.46**
Henicosapentaenoic acid, mg	14.6 (8.9)	8.9 (7.2)	-39.0**	0.21	0.32*	10.9 (7.2)	9.3 (7.1)	-15.5*	0.29	0.43**
Docosadienoic acid, mg	1.2 (0.9)	0.6 (0.4)	-52.9**	0.04	0.07	1.0 (0.7)	0.6 (0.4)	-39.1**	0.20	0.37*
Docosatetraenoic acid, mg	23.1 (7.9)	17.0 (12.4)	-26.4**	0.44	0.53**	16.8 (5.9)	12.9 (7.2)	-23.1**	0.32	0.37**
Docosapentaenoic acid (n-3), mg	126 (60)	87 (58)	-30.9**	0.29	0.37**	91 (46)	84 (56)	-7.5	0.32	0.41**
Docosapentaenoic acid (n-6), mg	35.3 (12.4)	28.3 (31.9)	-20.0*	0.26	0.32*	27.8 (10.3)	20.7 (11.5)	-25.3**	0.23	0.28**
Docosahexaenoic acid, mg	768 (372)	490 (333)	-36.1**	0.23	0.31*	553 (284)	477 (325)	-13.7*	0.32	0.44**

12d-WFR, 12-day weighed food record; CC, correlation coefficient; FFQ, food frequency questionnaire; SD, standard deviation.

[a] Percentage differences: (FFQ-12d-WFR)/12d-WFR × 100 (%).*P*-values refer to paired t-test between intakes by short-FFQ and those by 12d-WFR for each; * P<0.05, ** P<0.01.

[b] Spearman's rank correlation coefficients based on energy-adjusted values.

[c] Spearman's rank correlation coefficients based on energy-adjusted values and expressed as deattenuated CC. * P<0.05, ** P<0.01.

[d] Deattenuated CCx = energy-adjusted CCx × SQRT(1 + λx/n), where λx is the ratio of within- to between-individual variance for nutrient x and n is the number of dietary records (12 days).

eTable 2. Daily intakes of energy and major nutrients according to the long-FFQ, and percentage differences between intakes by the long-FFQ and 12d-WFR and their correlations in men and women aged 40–74 years

	Men (n=98)				Yokoyama et al. [a]	Women (n=142)				Yokoyama et al. [a]
	12d-WFR Mean (SD)	Long-FFQ Mean (SD)	diff% [b]	CC [c,d]	CC [c,d]	12d-WFR Mean (SD)	Long-FFQ Mean (SD)	diff% [b]	CC [c,d]	CC [c,d]
Energy, kcal	2,199 (424)	2,283 (661)	3.8	0.45**	0.45	1,716 (295)	1,940 (637)	13.0**	0.18*	0.17
Water, g	2,703 (644)	2,912 (1,089)	7.7*	0.33**	0.32	2,329 (550)	2,663 (1,009)	14.3**	0.48**	0.48
Protein, g	84.2 (18.3)	79.2 (32.0)	-5.9	0.39**	0.40	70.4 (15.0)	77.1 (30.1)	9.5*	0.33**	0.33
Sum of amino acid residues [e], g	72.9 (16.0)	68.4 (28.3)	-6.1	0.40**	0.36	61.0 (13.2)	66.3 (26.1)	8.6*	0.32**	0.37
Total fat, g	63.3 (16.9)	60.9 (29.2)	-3.8	0.50**	0.53	54.9 (14.1)	64.1 (29.6)	16.7**	0.33**	0.33
Total fat in % energy	25.8 (4.2)	23.4 (6.4)	-9.2**	0.44**	0.46	28.6 (4.1)	28.8 (6.1)	0.4	0.33**	0.34
Saturated fatty acid, g	17.85 (5.60)	17.66 (9.32)	-1.0	0.46**	0.48	16.00 (5.08)	19.27 (10.55)	20.4**	0.42**	0.46
Monounsaturated fatty acid, g	23.68 (6.75)	23.00 (11.37)	-2.9	0.53**	0.55	20.01 (5.30)	23.84 (11.14)	19.1**	0.23*	0.21
Polyunsaturated fatty acid, g	14.06 (3.65)	13.53 (6.76)	-3.8	0.49**	0.50	12.23 (3.12)	13.87 (6.28)	13.4**	0.32**	0.28
n-3 polyunsaturated fatty acid, g	3.14 (1.08)	2.66 (1.41)	-15.2*	0.45**	0.38	2.51 (0.80)	2.80 (1.42)	11.7*	0.44**	0.40
n-6 polyunsaturated fatty acid, g	10.87 (2.99)	10.82 (5.52)	-0.4	0.46**	0.44	9.63 (2.56)	11.01 (5.02)	14.4**	0.32**	0.28
Triacylglycerol equivalents [f], g	58.2 (15.9)	56.6 (27.2)	-2.8	0.49**	0.49	50.6 (13.2)	59.5 (27.5)	17.6**	0.32**	0.33
Cholesterol, mg	348.2 (108.3)	316.8 (305.4)	-9.0	0.52**	0.53	287.5 (83.2)	296.0 (206.5)	3.0	0.37**	0.38
Carbohydrate, g	300.0 (63.1)	311.7 (101.3)	3.9	0.73**	0.74	248.7 (40.4)	278.6 (87.4)	12.0**	0.39**	0.40
Total dietary fiber [g], g	16.4 (5.7)	13.8 (6.8)	-15.9**	0.66**	0.66	16.2 (5.3)	17.2 (8.5)	6.2	0.60**	0.61
Water-soluble dietary fiber [h], g	3.5 (1.2)	3.1 (1.7)	-10.3*	0.61**	0.58	3.6 (1.4)	4.0 (2.1)	12.3**	0.58**	0.60
Water-insoluble dietary fiber [i], g	12.2 (4.4)	10.1 (4.9)	-17.1**	0.69**	0.68	12.0 (3.8)	12.6 (6.1)	5.0	0.60**	0.60
Sodium, mg	4,606 (1,094)	4,326 (1,998)	-6.1	0.34**	0.34	3,842 (927)	4,485 (2,059)	16.7**	0.36**	0.38
Potassium, mg	3,105 (889)	3,155 (1,306)	1.6	0.48**	0.48	2,972 (803)	3,631 (1,596)	22.2**	0.54**	0.54
Calcium, mg	565 (182)	582 (384)	3.0	0.56**	0.58	586 (203)	741 (472)	26.4**	0.42**	0.42
Magnesium, mg	331 (89)	360 (136)	8.5*	0.39**	0.39	299 (81)	364 (146)	21.5**	0.53**	0.51
Phosphorus, mg	1,230 (289)	1,235 (506)	0.4	0.47**	0.48	1,069 (247)	1,265 (533)	18.3**	0.36**	0.37
Iron, mg	9.5 (2.5)	9.7 (4.1)	2.2	0.55**	0.53	8.8 (2.6)	9.7 (3.8)	10.4**	0.60**	0.57
Zinc, mg	9.6 (2.3)	9.2 (3.1)	-4.5	0.42**	0.44	7.8 (1.6)	8.6 (3.1)	9.5**	0.34**	0.27
Copper, mg	1.42 (0.36)	1.39 (0.49)	-2.0	0.65**	0.64	1.24 (0.29)	1.36 (0.49)	10.4**	0.53**	0.49
Manganese, mg	4.85 (1.61)	4.72 (1.99)	-2.8	0.60**	0.58	4.47 (1.49)	4.54 (1.85)	1.5	0.75**	0.74
Iodine, µg	1893 (3149)	220 (195)	-88.4**	-0.06	0.01	1,741 (3,292)	269 (243)	-84.6**	0.21*	0.14
Selenium, µg	93 (24)	90 (43)	-2.9	0.21	0.15	73 (17)	87 (38)	18.5**	0.27*	0.15
Chromium, µg	11 (3)	9 (5)	-18.9**	0.44**	0.39	9 (3)	9 (5)	1.7	0.31**	0.26
Molybdenum, µg	245 (72)	265 (88)	8.2**	0.69**	0.64	201 (56)	243 (102)	21.1**	0.60**	0.57
Retinol, µg	300 (350)	373 (385)	24.5	0.42**	0.30	230 (182)	370 (481)	61.1**	0.47*	0.30
Alpha-carotene, µg	617 (368)	529 (525)	-14.3	0.34**	0.34	545 (315)	781 (1,211)	43.2*	0.55**	0.56
Beta-carotene, µg	3,663 (1,703)	2,921 (2,285)	-20.2**	0.55**	0.55	3,682 (1,549)	4,286 (3,775)	16.4*	0.52**	0.50
Cryptoxanthin, µg	308 (338)	600 (541)	95.0**	0.63**	0.57	432 (343)	1,329 (1,580)	208.1**	0.40**	0.37
Beta carotene equivalents, µg	4,305 (1,988)	3,498 (2,613)	-18.7**	0.53**	0.53	4,313 (1,776)	5,348 (4,550)	24.0**	0.52**	0.51
Retinol equivalents, µg	665 (383)	662 (490)	-0.4	0.46**	0.35	595 (267)	813 (665)	36.7**	0.50**	0.41
Vitamin D, µg	11.6 (5.4)	10.4 (6.9)	-10.7	0.36**	0.32	9.1 (5.1)	10.6 (7.4)	16.3*	0.53**	0.49
Alpha-tocopherol, mg	8.9 (2.7)	8.2 (4.2)	-8.0	0.52**	0.52	8.4 (2.7)	9.4 (4.6)	11.5*	0.52**	0.50
Beta-tocopherol, mg	0.4 (0.1)	0.4 (0.2)	5.4	0.51**	0.54	0.3 (0.1)	0.4 (0.2)	19.1**	0.24*	0.24
Gamma-tocopherol, mg	11.5 (3.5)	11.0 (6.1)	-4.8	0.29**	0.28	10.6 (3.2)	12.2 (6.4)	15.3**	0.31**	0.29
Delta-tocopherol, mg	3.1 (1.1)	2.7 (1.9)	-13.4*	0.54**	0.56	2.9 (1.0)	2.9 (1.6)	0.6	0.51**	0.53
Vitamin K, µg	302.1 (133.2)	273.7 (165.4)	-9.4	0.55**	0.56	301.6 (115.2)	348.5 (239.4)	15.6**	0.51**	0.52
Vitamin B_1, mg	1.30 (0.53)	1.06 (0.42)	-19.0**	0.36**	0.35	1.05 (0.36)	1.13 (0.46)	7.1	0.35**	0.36
Vitamin B_2, mg	1.65 (0.62)	1.45 (0.74)	-12.3**	0.42**	0.43	1.46 (0.44)	1.64 (0.80)	11.9**	0.43**	0.43
Niacin, mg	23.9 (6.9)	25.3 (10.0)	5.5	0.29**	0.28	18.5 (5.4)	22.9 (9.0)	23.9**	0.31**	0.32
Vitamin B_6, mg	1.90 (0.93)	1.68 (0.65)	-11.9**	0.41**	0.42	1.50 (0.58)	1.64 (0.68)	9.8*	0.59**	0.59
Vitamin B_{12}, µg	9.7 (4.2)	8.2 (4.9)	-15.5**	0.43**	0.34	7.3 (3.4)	8.0 (4.9)	9.5	0.43**	0.35
Folate, µg	457 (161)	405 (206)	-11.4**	0.63**	0.63	449 (148)	484 (238)	7.7*	0.61**	0.62
Pantothenic acid, mg	6.82 (1.79)	7.33 (3.00)	7.4	0.62**	0.62	6.10 (1.54)	7.82 (3.39)	28.2**	0.47**	0.46
Biotin, µg	51 (14)	61 (32)	20.4**	0.34**	0.17	45 (12)	60 (26)	32.1**	0.38**	0.36
Vitamin C, mg	142 (70)	125 (87)	-12.2*	0.64**	0.65	156 (72)	185 (112)	19.1**	0.63**	0.66
Daidzein, mg	13.90 (8.51)	17.01 (16.63)	22.4*	0.61**	0.63	13.16 (7.30)	17.12 (12.35)	30.1**	0.54**	0.55
Genistein, mg	23.38 (14.31)	27.92 (27.90)	19.4*	0.61**	0.63	22.31 (12.51)	28.22 (20.70)	26.5**	0.52**	0.53
Ethanol, g	27.1 (24.1)	35.3 (31.0)	30.6**	0.84**	0.82	4.7 (7.9)	4.4 (9.0)	-7.6	0.66**	0.67
Lycopene, mg	2,035.7 (2,231.2)	6,059.1 (12,728.1)	197.6**	0.36**	NA	1,872.7 (1,418.6)	4,744.2 (5,272.5)	153.3**	0.44**	NA

[a] Yokoyama et al. Validity of Short and Long Self-Administered Food Frequency Questionnaires in Ranking Dietary Intake in Middle-Aged and Elderly Japanese in the Japan Public Health Center-Based Prospective Study for the Next Generation (JPHC-NEXT) Protocol Area. J Epidemiol. 2016; 26:420-32.

12d-WFR, 12-day weighed food record; CC, correlation coefficient; FFQ, food frequency questionnaire; SD, standard deviation; NA, not applicable.

[b] Percentage differences: (FFQ-12-day WFR)/12-day WFR × 100 (%). P-values refer to the paired t-test between intakes by the long-FFQ and those by 12-day WFR for each; * P<0.05, ** P<0.01.

[c] Spearman's rank correlation coefficients based on energy-adjusted values (other than energy intake and total fat in % energy) and expressed as deattenuated CC. * P<0.05, ** P<0.01.

[d] Deattenuated CCx = energy-adjusted CCx × SQRT(1 + λx/n), where λx is the ratio of within- to between-individual variance for nutrient x, and n is the number of dietary records (12 days).

[e] 411 food items were substituted with the value of protein because there were unmeasured values for the sum of amino acid residues.

[f] 423 food items were substituted with the value of fat because there were unmeasured values for triacylglycerol equivalents.

[g] Total dietary fiber was measured by the Prosky or modified Prosky method.

[h] Water-soluble dietary fiber measured by the Prosky or modified Prosky method.

[i] Water-insoluble dietary fiber measured by the Prosky or modified Prosky method.

eTable 3. Daily intakes of energy and major nutrients according to the short-FFQ, and percentage differences between intakes by the short-FFQ and 12d-WFR and their correlations in men and women aged 40–74 years

	Men (n=92)				**Yokoyama et al.** [a]	Women (n=136)				**Yokoyama et al.** [a]
	12d-WFR Mean (SD)	Short-FFQ Mean (SD)	diff% [b]	CC [c,d]	CC [c,d]	12d-WFR Mean (SD)	Short-FFQ Mean (SD)	diff% [b]	CC [c,d]	CC [c,d]
Energy, kcal	2,227 (417)	1,779 (495)	-20.1**	0.49**	0.49	1,720 (295)	1,321 (365)	-23.2**	0.16	0.16
Water, g	2,747 (615)	2,309 (923)	-15.9**	0.42**	0.42	2,343 (535)	1,889 (742)	-19.4**	0.57**	0.58
Protein, g	85.3 (17.9)	61.2 (24.5)	-28.3**	0.33**	0.35	70.6 (15.0)	53.6 (18.9)	-24.1**	0.42**	0.43
Sum of amino acid residues [e], g	73.9 (15.6)	52.4 (21.4)	-29.1**	0.37**	0.31	61.2 (13.2)	45.4 (16.1)	-25.8**	0.41**	0.43
Total fat, g	64.6 (16.4)	42.3 (22.6)	-34.5**	0.48**	0.50	55.1 (14.3)	38.0 (17.7)	-31.0**	0.28**	0.29
Total fat in % energy	26.0 (4.0)	20.8 (7.3)	-20.1**	0.47**	0.49	28.6 (4.1)	25.0 (6.3)	-12.5**	0.13	0.14
Saturated fatty acid, g	18.20 (5.48)	12.40 (7.19)	-31.8**	0.47**	0.47	16.04 (5.18)	10.83 (5.70)	-32.5**	0.41**	0.44
Monounsaturated fatty acid, g	24.19 (6.58)	15.68 (8.64)	-35.2**	0.46**	0.45	20.08 (5.40)	14.20 (7.12)	-29.3**	0.22*	0.21
Polyunsaturated fatty acid, g	14.37 (3.52)	9.48 (5.33)	-34.0**	0.51**	0.48	12.28 (3.17)	8.69 (3.85)	-29.3**	0.26**	0.24
n-3 polyunsaturated fatty acid, g	3.21 (1.07)	2.01 (1.17)	-37.4**	0.42**	0.36	2.53 (0.81)	1.96 (1.03)	-22.3**	0.34**	0.30
n-6 polyunsaturated fatty acid, g	11.12 (2.90)	7.44 (4.29)	-33.1**	0.48**	0.44	9.66 (2.60)	6.68 (2.98)	-30.8**	0.26**	0.24
Triacylglycerol equivalents [f], g	59.4 (15.4)	39.1 (21.0)	-34.1**	0.47**	0.44	50.8 (13.4)	35.1 (16.4)	-30.8**	0.29**	0.31
Cholesterol, mg	352.2 (106.1)	270.5 (258.7)	-23.2**	0.43**	0.45	286.9 (82.4)	199.8 (101.4)	-30.3**	0.43**	0.46
Carbohydrate, g	302.1 (63.8)	236.7 (72.8)	-21.6**	0.68**	0.68	249.2 (39.4)	195.4 (50.6)	-21.6**	0.45**	0.44
Total dietary fiber [g], g	16.6 (5.7)	8.8 (4.4)	-47.2**	0.62**	0.65	16.3 (5.2)	10.5 (5.0)	-35.7**	0.56**	0.57
Water-soluble dietary fiber [h], g	3.5 (1.2)	1.8 (1.1)	-48.0**	0.60**	0.62	3.6 (1.4)	2.4 (1.2)	-34.3**	0.54**	0.54
Water-insoluble dietary fiber [i], g	12.4 (4.3)	6.5 (3.1)	-47.2**	0.62**	0.64	12.1 (3.7)	7.7 (3.6)	-36.2**	0.58**	0.59
Sodium, mg	4,657 (1,065)	2,888 (1,403)	-38.0**	0.47**	0.50	3,834 (915)	2,817 (1,264)	-26.5**	0.37**	0.39
Potassium, mg	3,150 (861)	2,180 (915)	-30.8**	0.44**	0.46	2,997 (780)	2,316 (944)	-22.7**	0.48**	0.47
Calcium, mg	572 (176)	346 (236)	-39.6**	0.54**	0.56	589 (204)	355 (187)	-39.8**	0.61**	0.60
Magnesium, mg	336 (87)	276 (121)	-17.8**	0.35**	0.34	301 (80)	260 (96)	-13.7**	0.46**	0.45
Phosphorus, mg	1246 (282)	907 (355)	-27.2**	0.45**	0.47	1,074 (246)	799 (278)	-25.6**	0.52**	0.54
Iron, mg	9.7 (2.5)	7.7 (3.6)	-20.3**	0.57**	0.56	8.8 (2.6)	7.2 (2.7)	-18.4**	0.66**	0.63
Zinc, mg	9.7 (2.3)	7.1 (2.4)	-27.0**	0.38**	0.40	7.9 (1.6)	6.0 (1.9)	-23.9**	0.40**	0.38
Copper, mg	1.43 (0.35)	1.04 (0.38)	-27.6**	0.57**	0.54	1.24 (0.29)	0.94 (0.30)	-24.1**	0.67**	0.65
Manganese, mg	4.94 (1.62)	3.62 (1.72)	-26.7**	0.50**	0.46	4.49 (1.48)	3.30 (1.27)	-26.5**	0.69**	0.68
Iodine, µg	1,674 (2,332)	167 (151)	-90.0**	0.16	0.18	1,634 (2,835)	191 (182)	-88.3**	0.23*	0.15
Selenium, µg	94 (23)	67 (34)	-28.5**	0.26*	0.24	73 (17)	60 (27)	-18.2**	0.41**	0.30
Chromium, µg	11 (3)	6 (5)	-44.8**	0.50**	0.38	9 (3)	5 (3)	-44.7**	0.36**	0.26
Molybdenum, µg	247 (72)	238 (92)	-3.6	0.54**	0.46	202 (56)	200 (63)	-1.1	0.68**	0.69
Retinol, µg	308 (358)	468 (527)	51.7**	0.43**	0.29	231 (184)	290 (373)	25.5	0.64**	0.34
Alpha-carotene, µg	631 (375)	495 (477)	-21.5*	0.54**	0.51	556 (317)	717 (592)	29.0**	0.37**	0.37
Beta-carotene, µg	3,734 (1,718)	2,334 (2,015)	-37.5**	0.51**	0.51	3,739 (1,545)	3,493 (2,454)	-6.6	0.39**	0.37
Cryptoxanthin, µg	313 (346)	681 (969)	117.7**	0.46**	0.42	436 (347)	1,257 (1,540)	188.2**	0.44**	0.40
Beta carotene equivalents, µg	4,382 (1,996)	2,920 (2,429)	-33.4**	0.46**	0.46	4,383 (1,769)	4,472 (2,962)	2.0	0.35**	0.34
Retinol equivalents, µg	680 (384)	711 (618)	4.5	0.54**	0.38	603 (267)	663 (472)	10.0	0.42**	0.34
Vitamin D, µg	11.8 (5.4)	9.6 (6.6)	-18.5**	0.48**	0.44	9.2 (5.1)	9.3 (6.0)	0.4	0.52**	0.47
Alpha-tocopherol, mg	9.1 (2.6)	5.0 (2.7)	-44.9**	0.42**	0.41	8.5 (2.7)	5.7 (3.0)	-32.8**	0.49**	0.49
Beta-tocopherol, mg	0.4 (0.1)	0.2 (0.1)	-42.5**	0.43**	0.53	0.3 (0.1)	0.2 (0.1)	-29.8**	0.34**	0.36
Gamma-tocopherol, mg	11.8 (3.4)	7.5 (5.0)	-36.9**	0.36**	0.35	10.6 (3.2)	7.2 (4.0)	-32.0**	0.26**	0.29
Delta-tocopherol, mg	3.2 (1.1)	2.2 (1.7)	-29.8**	0.54**	0.57	2.9 (1.0)	2.1 (1.2)	-26.7**	0.50**	0.53
Vitamin K, µg	306.9 (133.4)	209.5 (154.2)	-31.8**	0.64**	0.65	304.2 (115.4)	257.8 (158.6)	-15.3**	0.53**	0.53
Vitamin B_1, mg	1.33 (0.53)	0.81 (0.38)	-38.9**	0.33**	0.31	1.06 (0.37)	0.78 (0.31)	-26.9**	0.37**	0.37
Vitamin B_2, mg	1.69 (0.61)	1.12 (0.58)	-33.4**	0.53**	0.54	1.47 (0.44)	1.08 (0.46)	-27.1**	0.62**	0.61
Niacin, mg	24.4 (6.8)	20.5 (8.9)	-16.0**	0.29*	0.29	18.6 (5.4)	18.4 (7.1)	-1.5	0.16	0.18
Vitamin B_6, mg	2.0 (0.9)	1.4 (0.6)	-30.2**	0.40**	0.41	1.5 (0.6)	1.2 (0.5)	-20.4**	0.44**	0.46
Vitamin B_{12}, µg	9.9 (4.2)	7.6 (5.0)	-22.7**	0.37**	0.28	7.3 (3.4)	7.0 (4.4)	-4.4	0.50**	0.46
Folate, µg	465 (159)	293 (170)	-37.1**	0.55**	0.55	453 (147)	313 (154)	-30.9**	0.54**	0.55
Pantothenic acid, mg	6.93 (1.75)	5.59 (2.20)	-19.4**	0.63**	0.64	6.14 (1.53)	5.14 (1.86)	-16.2**	0.60**	0.61
Biotin, µg	52 (13)	50 (25)	-2.5	0.39**	0.32	46 (12)	43 (16)	-5.0	0.35**	0.32
Vitamin C, mg	145 (70)	67 (55)	-54.0**	0.55**	0.56	157 (72)	97 (65)	-38.2**	0.56**	0.59
Daidzein, mg	14.27 (8.62)	17.07 (18.48)	19.6	0.63**	0.65	13.31 (7.39)	15.61 (11.12)	17.3**	0.63**	0.64
Genistein, mg	23.99 (14.49)	27.47 (29.97)	14.5	0.63**	0.64	22.57 (12.66)	25.52 (19.04)	13.0*	0.62**	0.63
Ethanol, g	27.8 (24.6)	NA	NA	NA	NA	4.7 (8.0)	NA	NA	NA	NA
Lycopene, mg	2,076.3 (2,282.1)	2,996.0 (5,229.0)	44.3	0.46**	NA	1,868.1 (1,405.2)	2,750.6 (3,429.6)	47.2**	0.41**	NA

[a] Yokoyama et al. Validity of Short and Long Self-Administered Food Frequency Questionnaires in Ranking Dietary Intake in Middle-Aged and Elderly Japanese in the Japan Public Health Center-Based Prospective Study for the Next Generation (JPHC-NEXT) Protocol Area. J Epidemiol. 2016; 26:420-32.
12d-WFR, 12-day weighed food record; CC, correlation coefficient; FFQ, food frequency questionnaire; SD, standard deviation; NA, not applicable.
[b] Percentage differences: (FFQ-12-day WFR)/12-day WFR × 100 (%).*P*-values refer to the paired t-test between intakes by the short-FFQ and those by 12-day WFR for each; * $P<0.05$, ** $P<0.01$.
[c] Spearman's rank correlation coefficients based on energy-adjusted values (other than energy intake and total fat in % energy) and expressed as deattenuated CC. * $P<0.05$, ** $P<0.01$.
[d] Deattenuated CCx = energy-adjusted CCx × SQRT(1 + λx/n), where λx is the ratio of within- to between-individual variance for nutrient x, and n is the number of dietary records (12 days).
[e] 411 food items were substituted with the value of protein because there were unmeasured values for sum of amino acid residues.
[f] 423 food items were substituted withthe value of fat because there were unmeasured values for triacylglycerol equivalents.
[g] Total dietary fiber was measured by the Prosky or modified prosky method.
[h] Water-soluble dietary fiber measured by the Prosky or modified Prosky method.
[i] Water-insoluble dietary fiber measured by the Prosky or modified Prosky method.

eTable 4. Daily intakes of energy and nutrients according to the FFQs and their intra-correlations among men and women aged 40–74 years

Intakes	Men (n=98)			Women (n=142)		
	Long-FFQ1 [a] Mean (SD)	Long-FFQ2 [b] Mean (SD)	ICC (95% CI) [c]	Long-FFQ1 [a] Mean (SD)	Long-FFQ2 [b] Mean (SD)	ICC (95% CI) [c]
**Sugars**						
Available carbohydrate; monosaccharide equivalents, g	328.0 (115.5)	322.4 (103.3)	0.63 (0.61–0.66)	284.4 (84.9)	281.5 (85.9)	0.45 (0.42–0.50)
Available carbohydrate; mass matter, g	302.8 (107.4)	297.1 (95.1)	0.63 (0.62–0.66)	263.2 (79.2)	260.6 (80.2)	0.45 (0.42–0.50)
Available carbohydrate; calculated by difference, g	313.4 (111.8)	307.2 (98.3)	0.64 (0.63–0.67)	272.5 (82.0)	270.2 (83.0)	0.46 (0.43–0.51)
Starch, g	203.2 (71.0)	206.8 (76.4)	0.52 (0.49–0.56)	166.3 (45.1)	162.1 (43.6)	0.52 (0.49–0.56)
Glucose, g	13.5 (13.5)	11.0 (7.2)	0.67 (0.65–0.69)	12.6 (8.5)	12.4 (8.6)	0.64 (0.62–0.66)
Fructose, g	14.5 (15.4)	11.5 (7.6)	0.60 (0.58–0.63)	15.2 (10.0)	15.4 (10.9)	0.65 (0.63–0.67)
Galactose, g	0.6 (1.3)	0.5 (1.3)	0.41 (0.36–0.48)	0.7 (1.2)	0.9 (1.6)	0.61 (0.59–0.63)
Sucrose, g	17.3 (15.7)	15.1 (10.6)	0.77 (0.77–0.78)	24.6 (15.4)	22.6 (14.6)	0.51 (0.49–0.55)
Maltose, g	0.9 (0.9)	0.8 (0.7)	0.67 (0.66–0.69)	1.0 (0.7)	0.9 (0.8)	0.58 (0.56–0.61)
Lactose, g	8.9 (12.2)	7.6 (9.7)	0.58 (0.55–0.61)	9.9 (11.7)	12.4 (14.0)	0.58 (0.56–0.61)
Trehalose, g	0.2 (0.2)	0.2 (0.2)	0.44 (0.40–0.51)	0.3 (0.2)	0.2 (0.2)	0.53 (0.50–0.56)
Total dietary fiber [d], g	19.7 (16.3)	17.7 (8.5)	0.51 (0.47–0.55)	22.3 (13.0)	20.7 (9.7)	0.63 (0.62–0.65)
Total dietary fiber (AOAC.2011.25) [e], g	6.2 (4.9)	5.8 (4.2)	0.50 (0.47–0.55)	5.9 (4.8)	5.4 (3.3)	0.41 (0.36–0.47)
Low-molecular-weight water-soluble dietary fiber [f], g	1.8 (1.5)	1.7 (1.3)	0.50 (0.47–0.55)	1.4 (0.9)	1.4 (0.8)	0.48 (0.45–0.53)
High-molecular-weight water-soluble dietary fiber [g], g	1.8 (1.4)	1.7 (1.3)	0.48 (0.44–0.53)	1.7 (1.1)	1.6 (0.9)	0.38 (0.34–0.45)
Water-insoluble dietary fiber [h], g	2.6 (2.1)	2.5 (1.7)	0.50 (0.47–0.55)	2.8 (3.1)	2.5 (1.6)	0.37 (0.33–0.44)
Resistant starch, g	0.6 (0.7)	0.4 (0.3)	0.61 (0.59–0.64)	0.5 (0.4)	0.5 (0.6)	0.61 (0.60–0.64)
Sorbitol, g	0.4 (0.5)	0.3 (0.3)	0.61 (0.59–0.64)	0.5 (0.5)	0.5 (0.4)	0.60 (0.58–0.63)
Mannitol, g	0.03 (0.03)	0.03 (0.03)	0.23 (0.16–0.41)	0.04 (0.04)	0.04 (0.03)	0.49 (0.46–0.53)
Acetic acid, g	0.1 (0.1)	0.1 (0.1)	0.38 (0.33–0.47)	0.1 (0.1)	0.1 (0.1)	0.55 (0.53–0.58)
Lactic acid, g	0.9 (1.7)	0.7 (1.3)	0.49 (0.45–0.54)	0.9 (1.2)	1.1 (1.4)	0.64 (0.63–0.66)
Gluconic acid, g	0.003 (0.017)	0.001 (0.003)	0.38 (0.33–0.46)	0.002 (0.005)	0.002 (0.004)	0.62 [i]
Oxalic acid, g	0.08 (0.07)	0.08 (0.09)	0.45 (0.41–0.51)	0.11 (0.14)	0.11 (0.14)	0.52 (0.49–0.55)
Succinic acid, g	0.013 (0.066)	0.010 (0.043)	0.56 (0.53–0.59)	0.005 (0.019)	0.005 (0.018)	0.74 (0.73–0.75)
Fumaric acid, g	0.004 (0.005)	0.004 (0.004)	0.55 (0.53–0.59)	0.006 (0.008)	0.005 (0.005)	0.42 (0.38–0.47)
Malic acid, g	0.5 (0.5)	0.4 (0.3)	0.61 (0.59–0.64)	0.7 (0.4)	0.7 (0.4)	0.55 (0.53–0.58)
Tartaric acid, g	0.08 (0.15)	0.05 (0.08)	0.43 (0.39–0.50)	0.06 (0.08)	0.06 (0.07)	0.45 (0.42–0.50)
Citric acid, g	1.0 (0.8)	0.8 (0.7)	0.55 (0.52–0.59)	1.2 (0.8)	1.2 (0.9)	0.62 (0.60–0.64)
Ferulic acid, mg	1.2 (1.2)	1.2 (1.2)	0.50 (0.47–0.55)	1.6 (1.8)	1.6 (1.7)	0.44 (0.40–0.49)
Chlorogenic acid, mg	0.1 (0.1)	0.1 (0.2)	0.52 (0.49–0.56)	0.1 (0.2)	0.1 (0.2)	0.53 (0.51–0.57)
Quinic acid, g	0.04 (0.05)	0.03 (0.03)	0.35 (0.30–0.45)	0.08 (0.15)	0.07 (0.08)	0.50 (0.47–0.54)
Median			0.52			0.53
**Amino acids**						
Isoleucine, mg	3,766 (3,140)	3,412 (1,479)	0.58 (0.56–0.61)	3,283 (1,781)	3,360 (1,372)	0.45 (0.41–0.50)
Leucine, mg	6,711 (5,451)	6,100 (2,567)	0.56 (0.54–0.60)	5,835 (3,094)	5,975 (2,406)	0.45 (0.41–0.50)
Lysine, mg	5,555 (5,528)	4,899 (2,306)	0.60 (0.57–0.63)	4,765 (2,715)	4,920 (2,203)	0.46 (0.42–0.50)
Methionine, mg	1,978 (1,796)	1,774 (761)	0.54 (0.51–0.58)	1,653 (835)	1,714 (708)	0.45 (0.42–0.50)
Cystine, mg	1,319 (899)	1,234 (485)	0.58 (0.55–0.61)	1,133 (556)	1,141 (404)	0.40 (0.35–0.46)
Sulfur-containing amino acids, mg	3,296 (2,678)	3,007 (1,233)	0.55 (0.52–0.59)	2,787 (1,377)	2,859 (1,103)	0.43 (0.39–0.48)
Phenylalanine, mg	3,950 (3,022)	3,626 (1,494)	0.58 (0.56–0.61)	3,472 (1,912)	3,507 (1,354)	0.45 (0.41–0.50)
Tyrosine, mg	3,204 (2,546)	2,925 (1,253)	0.56 (0.53–0.59)	2,794 (1,528)	2,866 (1,162)	0.44 (0.40–0.49)
Aromatic amino acids, mg	7,176 (5,601)	6,573 (2,765)	0.57 (0.54–0.60)	6,283 (3,453)	6,394 (2,525)	0.44 (0.40–0.49)
Threonine, mg	3,637 (3,236)	3,275 (1,424)	0.59 (0.57–0.62)	3,139 (1,689)	3,203 (1,314)	0.46 (0.43–0.51)
Tryptophan, mg	1,061 (849)	971 (409)	0.57 (0.55–0.61)	930 (499)	947 (375)	0.46 (0.42–0.51)
Valine, mg	4,547 (3,654)	4,139 (1,729)	0.55 (0.53–0.59)	3,953 (2,052)	4,056 (1,620)	0.45 (0.41–0.50)
Histidine, mg	2,860 (2,731)	2,539 (1,077)	0.58 (0.56–0.61)	2,425 (1,403)	2,439 (1,037)	0.47 (0.44–0.52)
Arginine, mg	5,314 (4,485)	4,870 (1,971)	0.61 (0.59–0.63)	4,617 (2,771)	4,564 (1,751)	0.47 (0.43–0.51)
Alanine, mg	4,372 (3,984)	3,945 (1,605)	0.59 (0.57–0.62)	3,696 (1,933)	3,730 (1,462)	0.48 (0.45–0.53)
Aspartic acid, mg	7,986 (6,793)	7,270 (3,108)	0.63 (0.61–0.65)	7,106 (4,193)	7,110 (2,855)	0.49 (0.46–0.53)
Glutamic acid, mg	16,055 (11,667)	14,730 (6,059)	0.62 (0.60–0.64)	14,292 (7,463)	14,377 (5,592)	0.50 (0.47–0.54)
Glycine, mg	3,947 (3,761)	3,540 (1,407)	0.62 (0.60–0.65)	3,330 (1,891)	3,290 (1,287)	0.47 (0.44–0.52)
Proline, mg	5,106 (3,670)	4,636 (1,982)	0.56 (0.53–0.60)	4,502 (2,312)	4,627 (1,953)	0.52 (0.49–0.55)
Serine, mg	4,437 (3,493)	4,073 (1,783)	0.57 (0.54–0.60)	3,879 (2,075)	3,961 (1,568)	0.44 (0.40–0.49)
Hydroxyproline, mg	316 (540)	252 (152)	0.54 (0.51–0.58)	218 (159)	216 (130)	0.58 (0.56–0.61)
Median			0.58			0.46

	Men (n=98)			Women (n=142)		
	Long-FFQ1 [a]	Long-FFQ2 [b]	ICC (95% CI) [c]	Long-FFQ1 [a]	Long-FFQ2 [b]	ICC (95% CI) [c]
	Mean (SD)	Mean (SD)		Mean (SD)	Mean (SD)	
**Fatty acids**						
Butyric acid, mg	267 (311)	250 (279)	0.52 (0.49–0.57)	310 (336)	384 (401)	0.53 (0.51–0.57)
Hexanoic acid, mg	167 (197)	156 (178)	0.55 (0.53–0.59)	191 (213)	238 (253)	0.56 (0.54–0.59)
Heptanoic acid, mg	1.6 (2.4)	1.4 (2.4)	0.48 (0.44–0.53)	1.9 (2.8)	2.5 (3.3)	0.59 (0.57–0.62)
Octanoic acid, mg	102 (116)	96 (104)	0.57 (0.54–0.60)	121 (127)	148 (151)	0.56 (0.54–0.59)
Decanoic acid, mg	242 (258)	223 (225)	0.55 (0.53–0.59)	272 (273)	331 (324)	0.57 (0.55–0.60)
Lauric acid, mg	346 (324)	318 (263)	0.61 (0.59–0.64)	398 (343)	465 (409)	0.58 (0.56–0.61)
Tridecanoic acid, mg	4.8 (7.1)	4.3 (7.1)	0.54 (0.51–0.58)	5.6 (8.5)	7.4 (9.9)	0.61 (0.60–0.64)
Myristic acid, mg	1,611 (1,916)	1,375 (927)	0.59 (0.57–0.62)	1,494 (1,094)	1,703 (1,287)	0.48 (0.45–0.52)
Pentadecanoic acid, mg	155 (177)	133 (97)	0.58 (0.55–0.61)	142 (113)	165 (133)	0.52 (0.49–0.55)
Ant-pentadecanoic acid, mg	40.8 (47.0)	38.3 (41.8)	0.52 (0.49–0.57)	46.3 (50.2)	57.6 (59.9)	0.58 (0.56–0.61)
Palmitic acid, mg	11,961 (14,559)	10,310 (5,212)	0.57 (0.54–0.60)	10,300 (5,747)	10,713 (5,313)	0.45 (0.41–0.50)
Iso-palmitic acid, mg	18.9 (21.9)	17.8 (20.2)	0.52 (0.49–0.57)	21.6 (24.3)	27.0 (29.0)	0.59 (0.57–0.62)
Heptadecanoic acid, mg	196 (290)	162 (89)	0.57 (0.55–0.61)	153 (93)	162 (97)	0.44 (0.40–0.49)
Ant-heptadecanoic acid, mg	38.8 (45.0)	36.4 (39.7)	0.55 (0.53–0.59)	44.8 (47.6)	55.4 (56.7)	0.58 (0.56–0.61)
Stearic acid, mg	4,897 (6,684)	4,192 (2,217)	0.61 (0.58–0.63)	4,293 (2,435)	4,425 (2,325)	0.44 (0.40–0.49)
Arachidic acid, mg	200 (251)	180 (87)	0.55 (0.53–0.59)	221 (236)	200 (102)	0.47 (0.44–0.52)
Behenic acid, mg	118 (131)	110 (79)	0.55 (0.52–0.59)	164 (398)	121 (101)	0.46 (0.42–0.51)
Lignoceric acid, mg	58.8 (58.8)	56.4 (39.3)	0.48 (0.44–0.53)	82.1 (203.6)	60.0 (51.7)	0.44 (0.41–0.49)
Decenoic acid, mg	22.5 (26.0)	21.0 (22.9)	0.53 (0.50–0.58)	25.2 (27.5)	31.4 (32.6)	0.59 (0.58–0.62)
Myristoleic acid, mg	154 (234)	122 (101)	0.55 (0.52–0.59)	111 (102)	130 (116)	0.51 (0.48–0.54)
Pentadecenoic acid, mg	0.1 (0.4)	0.1 (0.1)	0.20 (0.13–0.42)	0.1 (0.1)	0.1 (0.1)	0.31 (0.25–0.40)
Palmitoleic acid, mg	1,247 (2,113)	993 (563)	0.55 (0.52–0.59)	864 (509)	899 (482)	0.40 (0.36–0.46)
Heptadecenoic acid, mg	141 (242)	113 (68)	0.57 (0.55–0.61)	98 (63)	102 (61)	0.46 (0.43–0.51)
Oleic acid, mg	8,445 (14,953)	6,909 (4,637)	0.56 (0.54–0.60)	5,852 (3,557)	6,257 (3,951)	0.37 (0.33–0.44)
Cis-vaccenic acid, mg	520 (1,025)	419 (266)	0.54 (0.51–0.58)	357 (215)	372 (209)	0.42 (0.39–0.48)
Icosenoic acid, mg	804 (1,655)	637 (366)	0.50 (0.46–0.55)	666 (497)	643 (377)	0.52 (0.49–0.55)
Docosenoic acid, mg	543 (1,383)	398 (316)	0.48 (0.44–0.53)	415 (370)	400 (322)	0.55 (0.53–0.58)
Tetracosenoic acid, mg	63.3 (137.3)	49.7 (30.9)	0.54 (0.51–0.58)	51.3 (36.9)	50.7 (30.9)	0.57 (0.55–0.60)
Hexadecadienoic acid, mg	17.0 (45.4)	12.1 (9.9)	0.55 (0.53–0.59)	11.7 (11.1)	11.7 (10.0)	0.54 (0.52–0.58)
Hexadecatrienoic acid, mg	14.6 (35.6)	10.6 (7.7)	0.51 (0.48–0.56)	11.3 (9.6)	11.3 (8.5)	0.56 (0.54–0.59)
Hexadecatetraenoic acid, mg	16.6 (44.9)	11.7 (9.7)	0.56 (0.53–0.59)	11.3 (10.9)	11.4 (9.6)	0.53 (0.51–0.57)
Linoleic acid, mg	12,045 (13,416)	10,563 (5,406)	0.60 (0.57–0.63)	11,791 (11,401)	10,792 (4,955)	0.51 (0.49–0.55)
α-linolenic acid, mg	1,810 (2,278)	1,584 (895)	0.61 (0.59–0.64)	1,826 (1,212)	1,756 (885)	0.49 (0.45–0.53)
γ-linolenic acid, mg	7.3 (15.2)	5.6 (4.3)	0.55 (0.52–0.59)	6.4 (5.5)	6.5 (5.0)	0.55 (0.52–0.58)
Octadecatetraenoic acid, mg	123 (322)	89 (73)	0.51 (0.47–0.55)	90.6 (82.6)	87.6 (74.0)	0.55 (0.53–0.58)
Icosadienoic acid, mg	80.7 (142.7)	66.2 (38.5)	0.50 (0.46–0.55)	58.9 (36.6)	60.2 (31.2)	0.55 (0.53–0.58)
Icosatrienoic acid (n-3), mg	2.3 (5.8)	1.7 (1.5)	0.40 (0.35–0.47)	1.8 (1.9)	1.7 (1.6)	0.44 (0.40–0.49)
Icosatrienoic acid (n-6), mg	42.6 (60.3)	35.8 (24.7)	0.50 (0.47–0.55)	30.9 (18.6)	34.6 (19.9)	0.33 (0.28–0.42)
Icosatetraenoic acid (n-3), mg	47.2 (104.1)	35.9 (25.4)	0.53 (0.50–0.57)	36.3 (32.1)	36.4 (26.9)	0.51 (0.48–0.54)
Arachidonic acid, mg	196 (268)	170 (147)	0.50 (0.46–0.55)	137 (90)	157 (102)	0.43 (0.39–0.48)
Icosapentaenoic acid, mg	419 (980)	311 (223)	0.54 (0.51–0.58)	310 (274)	305 (226)	0.59 (0.58–0.62)
Henicosapentaenoic acid, mg	14.7 (39.1)	10.5 (8.5)	0.55 (0.52–0.59)	10.3 (9.7)	10.3 (8.6)	0.54 (0.52–0.58)
Docosadienoic acid, mg	0.9 (2.0)	0.6 (0.5)	0.40 (0.35–0.48)	0.7 (0.6)	0.7 (0.5)	0.44 (0.40–0.49)
Docosatetraenoic acid, mg	22.3 (35.8)	19.2 (14.0)	0.46 (0.43–0.52)	15.1 (10.1)	16.5 (10.0)	0.53 (0.50–0.56)
Docosapentaenoic acid (n-3), mg	126.2 (270.1)	96.5 (63.2)	0.52 (0.49–0.57)	93.1 (76.8)	94.2 (64.9)	0.55 (0.52–0.58)
Docosapentaenoic acid (n-6), mg	33.7 (50.1)	30.6 (34.8)	0.50 (0.47–0.55)	22.4 (19.4)	27.0 (24.3)	0.50 (0.47–0.54)
Docosahexaenoic acid, mg	711 (1,574)	536 (361)	0.48 (0.44–0.53)	522 (446)	520 (368)	0.54 (0.52–0.58)
Median			0.54			0.53

	Men (n=98)			Women (n=142)		
	Long-FFQ1 [a]	Long-FFQ2 [b]	ICC (95% CI) [c]	Long-FFQ1 [a]	Long-FFQ2 [b]	ICC (95% CI) [c]
	Mean (SD)	Mean (SD)		Mean (SD)	Mean (SD)	
**Energy and main nutrients**						
Energy, kcal	2,397 (1,322)	2,283 (661)	0.27 (0.20–0.41)	1,947 (726)	1,940 (637)	0.61 (0.59–0.63)
Water, g	2,932 (1,374)	2,912 (1,089)	0.48 (0.45–0.54)	2,628 (956)	2,663 (1,009)	0.66 (0.65–0.68)
Protein, g	87.1 (71.5)	79.2 (32.0)	0.60 (0.57–0.63)	76.1 (39.6)	77.1 (30.1)	0.46 (0.42–0.51)
Sum of amino acid residues, g	75.1 (61.6)	68.4 (28.3)	0.58 (0.56–0.61)	65.4 (35.0)	66.3 (26.1)	0.44 (0.41–0.49)
Total fat, g	70.2 (89.1)	60.9 (29.2)	0.61 (0.59–0.64)	64.0 (39.7)	64.1 (29.6)	0.47 (0.44–0.52)
Total fat in % energy	23.6 (8.6)	23.4 (6.4)	0.48 (0.44–0.53)	28.2 (6.8)	28.8 (6.1)	0.54 (0.51–0.57)
Saturated fatty acid, g	20.43 (24.52)	17.66 (9.32)	0.57 (0.55–0.61)	18.27 (10.72)	19.27 (10.55)	0.48 (0.44–0.52)
Monounsaturated fatty acid, g	26.33 (36.17)	23.00 (11.37)	0.58 (0.56–0.62)	23.88 (14.98)	23.84 (11.14)	0.41 (0.37–0.47)
Polyunsaturated fatty acid, g	15.66 (19.41)	13.53 (6.76)	0.59 (0.57–0.62)	14.91 (12.92)	13.87 (6.28)	0.50 (0.47–0.54)
n-3 polyunsaturated fatty acid, g	3.25 (5.52)	2.66 (1.41)	0.56 (0.53–0.60)	2.88 (1.84)	2.80 (1.42)	0.49 (0.46–0.53)
n-6 polyunsaturated fatty acid, g	12.35 (13.86)	10.82 (5.52)	0.58 (0.56–0.61)	11.98 (11.41)	11.01 (5.02)	0.50 (0.47–0.54)
Triacylglycerol equivalents, g	65.2 (83.1)	56.6 (27.2)	0.61 (0.59–0.64)	59.6 (37.9)	59.5 (27.5)	0.47 (0.44–0.52)
Cholesterol, mg	339.2 (398.3)	316.8 (305.4)	0.52 (0.49–0.57)	250.3 (175.9)	296.0 (206.5)	0.46 (0.42–0.50)
Carbohydrate, g	318.6 (113.8)	311.7 (101.3)	0.63 (0.62–0.66)	282.7 (88.0)	278.6 (87.4)	0.51 (0.48–0.54)
Total dietary fiber [j], g	15.6 (14.4)	13.8 (6.8)	0.54 (0.52–0.58)	18.5 (10.9)	17.2 (8.5)	0.64 (0.63–0.66)
Water-soluble dietary fiber [k], g	3.6 (3.3)	3.1 (1.7)	0.55 (0.52–0.59)	4.3 (2.7)	4.0 (2.1)	0.62 (0.60–0.64)
Water-insoluble dietary fiber [l], g	11.5 (10.7)	10.1 (4.9)	0.55 (0.53–0.59)	13.6 (8.1)	12.6 (6.1)	0.64 (0.63–0.66)
Sodium, mg	5,021 (5,288)	4,326 (1,998)	0.65 (0.63–0.67)	4,553 (2,359)	4,485 (2,059)	0.67 (0.66–0.69)
Potassium, mg	3,495 (2,991)	3,155 (1,306)	0.67 (0.65–0.69)	3,614 (1,753)	3,631 (1,596)	0.67 (0.66–0.69)
Calcium, mg	651 (539)	582 (384)	0.57 (0.54–0.60)	685 (457)	741 (472)	0.67 (0.65–0.68)
Magnesium, mg	382 (258)	360 (136)	0.64 (0.63–0.67)	370 (200)	364 (146)	0.60 (0.58–0.63)
Phosphorus, mg	1,342 (961)	1,235 (506)	0.50 (0.47–0.55)	1,221 (605)	1,265 (533)	0.50 (0.47–0.54)
Iron, mg	10.5 (9.2)	9.7 (4.1)	0.68 (0.66–0.69)	10.0 (5.6)	9.7 (3.8)	0.60 (0.58–0.62)
Zinc, mg	9.9 (6.9)	9.2 (3.1)	0.47 (0.44–0.53)	8.5 (3.8)	8.6 (3.1)	0.44 (0.41–0.49)
Copper, mg	1.45 (0.86)	1.39 (0.49)	0.64 (0.62–0.66)	1.44 (0.80)	1.36 (0.49)	0.44 (0.40–0.49)
Manganese, mg	4.92 (3.23)	4.72 (1.99)	0.71 (0.70–0.73)	4.70 (2.09)	4.54 (1.85)	0.68 (0.67–0.69)
Iodine, µg	260 (353)	220 (195)	0.51 (0.48–0.56)	250 (224)	269 (243)	0.52 (0.49–0.55)
Selenium, µg	105 (110)	90 (43)	0.53 (0.51–0.58)	84 (46)	87 (38)	0.49 (0.46–0.53)
Chromium, µg	10 (9)	9 (5)	0.55 (0.52–0.59)	10 (5)	9 (5)	0.49 (0.45–0.53)
Molybdenum, µg	274 (138)	265 (88)	0.67 (0.66–0.69)	255 (180)	243 (102)	0.39 (0.35–0.45)
Retinol, µg	458 (721)	373 (385)	0.44 (0.40–0.50)	289 (304)	370 (481)	0.39 (0.34–0.45)
Alpha-carotene, µg	507 (495)	529 (525)	0.53 (0.50–0.57)	818 (1,151)	781 (1,211)	0.57 (0.55–0.60)
Beta-carotene, µg	3,393 (5,543)	2,921 (2,285)	0.58 (0.55–0.61)	4,509 (3,943)	4,286 (3,775)	0.63 (0.61–0.65)
Cryptoxanthin, µg	834 (1172)	600 (541)	0.57 (0.55–0.61)	1,442 (1,546)	1,329 (1,580)	0.53 (0.51–0.57)
Beta carotene equivalents, µg	4,071 (5,789)	3,498 (2,613)	0.57 (0.55–0.61)	5,640 (4,599)	5,348 (4,550)	0.62 (0.60–0.64)
Retinol equivalents, µg	795 (1124)	662 (490)	0.53 (0.50–0.57)	756 (513)	813 (665)	0.46 (0.43–0.51)
Vitamin D, µg	12.6 (20.6)	10.4 (6.9)	0.50 (0.46–0.55)	10.3 (10.1)	10.6 (7.4)	0.50 (0.47–0.54)
Alpha-tocopherol, mg	9.0 (10.8)	8.2 (4.2)	0.56 (0.54–0.60)	9.9 (6.9)	9.4 (4.6)	0.47 (0.44–0.52)
Beta-tocopherol, mg	0.4 (0.4)	0.4 (0.2)	0.35 (0.30–0.45)	0.4 (0.3)	0.4 (0.2)	0.48 (0.45–0.53)
Gamma-tocopherol, mg	13.0 (16.6)	11.0 (6.1)	0.55 (0.52–0.59)	13.3 (11.1)	12.2 (6.4)	0.48 (0.45–0.52)
Delta-tocopherol, mg	3.2 (3.6)	2.7 (1.9)	0.58 (0.56–0.62)	3.2 (3.3)	2.9 (1.6)	0.50 (0.47–0.54)
Vitamin K, µg	327.6 (457.6)	273.7 (165.4)	0.63 (0.61–0.66)	368.8 (377.1)	348.5 (239.4)	0.52 (0.49–0.56)
Vitamin B_1, mg	1.18 (1.13)	1.06 (0.42)	0.38 (0.33–0.46)	1.14 (0.51)	1.13 (0.46)	0.53 (0.51–0.57)
Vitamin B_2, mg	1.63 (1.40)	1.45 (0.74)	0.48 (0.44–0.53)	1.53 (0.78)	1.64 (0.80)	0.53 (0.51–0.57)
Niacin, mg	27.2 (22.9)	25.3 (10.0)	0.49 (0.46–0.55)	23.6 (13.4)	22.9 (9.0)	0.55 (0.53–0.58)
Vitamin B_6, mg	1.87 (1.72)	1.68 (0.65)	0.56 (0.54–0.60)	1.67 (0.82)	1.64 (0.68)	0.59 (0.57–0.62)
Vitamin B_{12}, µg	10.4 (17.3)	8.2 (4.9)	0.59 (0.57–0.63)	7.6 (5.6)	8.0 (4.9)	0.55 (0.52–0.58)
Folate, µg	463 (490)	405 (206)	0.67 (0.65–0.69)	488 (269)	484 (238)	0.72 (0.71–0.73)
Pantothenic acid, mg	8.00 (5.46)	7.33 (3.00)	0.50 (0.47–0.55)	7.68 (3.97)	7.82 (3.39)	0.51 (0.48–0.55)
Biotin, µg	64 (45)	61 (32)	0.53 (0.50–0.57)	59 (44)	60 (26)	0.39 (0.35–0.46)
Vitamin C, mg	151 (173)	125 (87)	0.67 (0.66–0.69)	190 (123)	185 (112)	0.69 (0.68–0.70)
Daidzein, mg	17.32 (16.67)	17.01 (16.63)	0.61 (0.59–0.64)	19.75 (30.75)	17.12 (12.35)	0.61 (0.59–0.63)
Genistein, mg	28.17 (27.33)	27.92 (27.90)	0.60 (0.58–0.63)	32.80 (52.25)	28.22 (20.70)	0.61 (0.59–0.63)
Ethanol, g	32.8 (30.6)	35.3 (31.0)	0.90 (0.89–0.90)	3.8 (8.2)	4.4 (9.0)	0.85 (0.84–0.85)
Lycopene, mg	5,035.2 (8,321.5)	6,059.1 (12,728.1)	0.42 (0.38–0.49)	5,090.0 (5,928.4)	4,744.2 (5,272.5)	0.45 (0.42–0.50)
Median			0.57			0.52

ICC, intraclass correlation coefficient; CI, confidence interval; FFQ, food frequency questionnaire; SD, standard deviation.
[a] The long-FFQ1 was conducted in November 2012.
[b] The long-FFQ2 was conducted from 1 year after the long-FFQ1 (November 2013).
[c] ICC was performed using the SAS MIXED procedure.
[d] Total dietary fiber was derived by combination of the AOAC.2011.25 method with either the Prosky or modified Prosky method.
[e] Total dietary fiber (AOAC.2011.25) measured using only the AOAC.2011.25 method.
[f] Low-molecular-weight water-soluble dietary fiber that remains soluble in 78% aqueous ethanol.
[g] High-molecular-weight water-soluble dietary fiber that precipitates from 78% aqueous ethanol.
[h] Water-insoluble dietary fiber measured using only the AOAC.2011.25 method.
[i] 95% CI not calculated due to the small number of measured food items and low intake.
[j] Total dietary fiber measured using the Prosky or modified Prosky method.
[k] Water-soluble dietary fiber measured using the Prosky or modified Prosky method.
[l] Water-insoluble dietary fiber measured using the Prosky or modified Prosky method.
